# App-Supported Lifestyle Interventions in Pregnancy to Manage Gestational Weight Gain and Prevent Gestational Diabetes: Scoping Review

**DOI:** 10.2196/48853

**Published:** 2023-11-10

**Authors:** Roxana Raab, Kristina Geyer, Sophia Zagar, Hans Hauner

**Affiliations:** 1 Institute of Nutritional Medicine, Else Kröner Fresenius Centre for Nutritional Medicine TUM School of Medicine and Health Technical University of Munich Munich Germany

**Keywords:** mobile health, mHealth, eHealth, mobile app, lifestyle intervention, pregnancy, gestational weight gain, gestational diabetes, prevention, overweight, obesity, mobile phone

## Abstract

**Background:**

Excessive gestational weight gain (GWG) and gestational diabetes mellitus (GDM) are common pregnancy complications that have been shown to be preventable through the use of lifestyle interventions. However, a significant gap exists between research on pregnancy lifestyle interventions and translation into clinical practice. App-supported interventions might aid in overcoming previous implementation barriers. The current status in this emerging research area is unknown.

**Objective:**

This scoping review aims to provide a comprehensive overview of planned, ongoing, and completed studies on eHealth and mobile health (mHealth) app–supported lifestyle interventions in pregnancy to manage GWG and prevent GDM. The review assesses the scope of the literature in the field; describes the population, intervention, control, outcomes, and study design (PICOS) characteristics of included studies as well as the findings on GWG and GDM outcomes; and examines app functionalities.

**Methods:**

The scoping review was conducted according to a preregistered protocol and followed established frameworks. Four electronic databases and 2 clinical trial registers were systematically searched. All randomized and quasi-randomized controlled trials (RCTs) of app-supported lifestyle interventions in pregnancy and related qualitative and quantitative research across the different study phases were considered for inclusion. Eligible studies and reports of studies were included until June 2022. Extracted data were compiled in descriptive analyses and reported in narrative, tabular, and graphical formats.

**Results:**

This review included 97 reports from 43 lifestyle intervention studies. The number of published reports has steadily increased in recent years; of the 97 included reports, 38 (39%) were trial register entries. Of the 39 identified RCTs, 10 efficacy or effectiveness trials and 8 pilot trials had published results on GWG (18/39, 46%); of these 18 trials, 7 (39%) trials observed significant intervention effects on GWG outcomes. Of all 39 RCTs, 5 (13%) efficacy or effectiveness trials reported GDM results, but none observed significant intervention effects on GDM. The RCTs included in the review were heterogeneous in terms of their PICOS characteristics. Most of the RCTs were conducted in high-income countries, included women with overweight or obesity and from all BMI categories, delivered multicomponent interventions, delivered interventions during pregnancy only, and focused on diet and physical activity. The apps used in the studies were mostly mHealth apps that included features for self-monitoring, feedback, goal setting, prompts, and educational content. Self-monitoring was often supported by wearable activity monitors and Bluetooth-connected weight scales.

**Conclusions:**

Research in this field is nascent, and the effectiveness and implementability of app-supported interventions have yet to be determined. The complexity and heterogeneity of intervention approaches pose challenges in identifying the most beneficial app features and intervention components and call for consistent and comprehensive intervention and outcome reporting.

## Introduction

### Background

Pregnancy is a sensitive period for the health of both mother and offspring. Almost half of all women enter pregnancy with overweight or obesity [1,2], increasing the risk of pregnancy complications such as excessive gestational weight gain (GWG) and gestational diabetes mellitus (GDM) [3-5]. Globally, approximately half of all pregnant women gain excessive weight during pregnancy [6], and 14% develop GDM [7]. Regardless of maternal prepregnancy BMI, these complications can negatively affect the short- and long-term health of mothers and their children. Mothers are at an increased risk for adverse pregnancy outcomes, postpartum weight retention, and metabolic disorders such as type 2 diabetes [5,8-10]. Children are susceptible for macrosomia or being large-​for-gestational-age at birth and developing obesity and its sequelae later in life [6,11-13]. These health consequences at the individual level are accompanied by immense costs to health care systems and societies [14].

Lifestyle is a modifiable determinant with the potential to improve maternal and child health. Recent systematic reviews and meta-analyses have summarized the effects of more than 3 decades of research on conventional, primarily face-to-face lifestyle intervention programs during pregnancy [15-21]. Overall, interventions moderately reduced the incidence of excessive GWG and postpartum weight retention and prevented GDM [15-17,20,21]. Interventions also reduced the risk for macrosomic births and large-for-gestational-age infants but did not influence long-term childhood weight outcomes [18,19]. Research investigating this lack of effect is ongoing [22]. Although the optimal intervention approach, including the most effective intervention components, delivery modes, and behavioral strategies, is still undetermined [15], researchers agree that lifestyle interventions should be implemented into practice to improve health outcomes and save health care costs [15,23]. However, implementing these interventions remains an unachieved goal [2,24]. While focusing on intervention efficacy and effectiveness primarily within controlled academic settings, research groups have neglected to apply implementation strategies and report on the outcomes of the implementation itself [24]. Moreover, barriers to implementation exist at the patient, provider, and system level, including lack of time, knowledge, and resources [25,26].

Recent advances and evolving opportunities for digital solutions open the way for innovative intervention approaches that could facilitate behavior change and overcome some of the implementation barriers. The most promising and convenient technology for providing evidence-based support for lifestyle behavior modification seems to be health apps, either accessible via mobile devices such as smartphones or tablet computers (mobile health [mHealth] apps) or via web browsers (eHealth apps). Their easy accessibility, scalability, and cost-effectiveness foster implementability [27]. Moreover, smartphones and apps are widely used across different age groups as well as socioeconomic and cultural backgrounds. App use has gained in popularity among young women; health information–seeking behavior has shifted from soliciting medical advice from health care providers to using digital media to obtain health information [28,29]. The potential of apps to offer on-demand lifestyle support with low resources and high reach is coupled with the ability to easily integrate various behavior change techniques (BCTs). These can be integrated into app features, such as self-monitoring, goal setting, or feedback, and have been shown to increase the effectiveness of interventions [30,31]. Moreover, real-time tracking of health and lifestyle data allows for the early identification of abnormal values and the tailoring of interventions according to one’s individual risk profile [32]. Thus, tailored support is possible on a large scale. Despite the increasing number of accessible lifestyle-related pregnancy apps on the market, few are of high quality, and many lack evidence-based resources and present information that is inconsistent with evidence-based guidelines, showing low use of BCTs and leaving uncertainties about data security [33-37].

The proven effectiveness of traditional lifestyle interventions on improving pregnancy outcomes and the urgent need to implement lifestyle interventions into clinical practice, together with the potential of apps to offer tailored, scalable, and evidence-based lifestyle support as well as a dearth of available high-quality apps, highlight the research and collaborative potential in this area. Indeed, studies on the development and integration of eHealth and mHealth apps to support a healthy lifestyle during pregnancy have emerged in the last years [38-42]. Previous systematic reviews and meta-analyses included studies using different technologies, such as SMS text messaging, telephone and video calls, websites, and social media. Only a few studies using apps were identified, with limited ability to draw conclusions based on the dearth of available evidence [43-47]. To the best of our knowledge, no scoping review mapping the current evidence of registered, ongoing, and completed pregnancy lifestyle intervention studies using eHealth and mHealth apps has been undertaken, which encouraged us to conduct this scoping review.

### Objectives

This review addresses 3 major research objectives: first, to assess the scope of the available literature in the field; second, to determine the concepts and characteristics of studies and their findings on GWG and GDM outcomes; and, third, to describe the functionalities of available eHealth and mHealth apps. On the basis of the evidence available, this review is further intended to identify knowledge gaps, inform ongoing and future intervention projects, and provide a basis for systematic reviews and meta-analyses.

## Methods

### Overview

This scoping review is based on a protocol that can be accessed through the Open Science Framework [48]. The review follows the framework developed by Arksey and O’Malley [49] and its refinement by Levac et al [50] as well as the JBI manual for evidence synthesis [51]. Accordingly, this work contains the following stages:

1. Identification of the research question
2. Identification of relevant studies
3. Selection of eligible studies
4. Charting of the data
5. Collating, summarizing, and reporting of the results

The optional stage 6, which includes consultation with stakeholders, is not covered by this review. The PRISMA-ScR (Preferred Reporting Items for Systematic Reviews and Meta-Analyses extension for Scoping Reviews) guides the reporting of this review [52] (Multimedia Appendix 1 [49-52]).

### Stage 1: Identification of the Research Question

This scoping review addresses the following research questions:

1. What is the scope of the literature that exists in the field of app-supported pregnancy lifestyle interventions to limit GWG and prevent GDM?
2. What are the concepts and characteristics of the available studies in terms of population, intervention, control, outcomes, and study design (PICOS), and what are the findings on GWG and GDM?
3. Which commercially available apps are being used in this field of research, and what kind of apps have been developed by researchers?

### Stage 2: Identification of Relevant Studies

The eligibility of the studies was assessed based on the population, concept, and context (PCC) framework suggested by JBI [51]. Table 1 provides an overview of the PCC criteria and the types of evidence considered in this scoping review. The population of interest was pregnant women and women who intend to become pregnant of any age and BMI category. The considered concept was the use of eHealth and mHealth apps within lifestyle intervention studies to manage GWG and prevent GDM in the context of pregnancy care and, at times combined with preconception care, postpartum care, or both in any country and any setting. Apps that were accessible through a mobile device, such as a smartphone or a tablet computer, were classified as mHealth apps, whereas those accessible through a web browser were classified as eHealth apps. Eligible reports were trial register entries (TREs), conference abstracts and further gray literature (eg, dissertations), study protocols, and full articles. Conference abstracts were excluded if a corresponding full text was available. Reviews, meta-analyses, and cohort studies without any references to pregnancy lifestyle interventions were not considered for inclusion in this review.

**Table 1:** Inclusion and exclusion criteria of this scoping review based on population, concept, context, and types of evidence.

	Inclusion criteria	Exclusion criteria
Population	Pregnant women and women who intend to become pregnant of any age and BMI category	Women with certain conditions or chronic diseases, such as type 1 or 2 diabetes mellitus and cancer
Concept	App-supported lifestyle intervention studies to limit GWG^a^ and prevent GDM^b^	eHealth studies involving SMS text messages, telephone or video calls, websites, and social media only
Context	Pregnancy care (+ preconception care + postpartum care), any setting, and any country (high-, middle-, and low-income countries)	N/A^c^
Types of evidence	Full articles, trial register entries, study protocols, gray literature (eg, conference abstracts), RCTs^d^ and quasi-RCTs, and related qualitative and quantitative investigations (eg, surveys, focus groups, and interviews)	Reviews and meta-analyses, editorial articles, and cohort studies

^a^GWG: gestational weight gain.

^b^GDM: gestational diabetes mellitus.

^c^N/A: not applicable.

^d^RCT: randomized controlled trial.

The PCC criteria guided the conduct of the search strategy. Preliminary searches in PubMed and Embase were performed to identify relevant keywords and controlled vocabulary to refine the search strategy. The final search strategy was adapted to fit the individual search criteria of each included database. No restrictions on publication status or language were applied. Hedges to filter for human studies were applied where appropriate. PubMed, Embase, Web of Science, and the Cochrane Library were searched on May 17, 2021 (Multimedia Appendix 2). The search was updated on November 11, 2021. The updated search only considered studies published after the initial search date or 2021, depending on the applicable custom range of each database. The International Clinical Trials Registry Platform and ClinicalTrials.gov were searched on May 30, 2022 (Multimedia Appendix 2). Complementarily, Google, Google Scholar, and PubMed were continually searched for relevant literature until June 2022. This involved searching for new studies as well as for reports of already identified studies. The reference lists of included articles and identified relevant reviews were additionally screened.

### Stage 3: Selection of Eligible Studies

All search hits were exported to the reference management software EndNote X9.3.3 (Clarivate). Duplicates were removed by 1 reviewer. The screening of titles, abstracts, and full texts was based on the PCC criteria and performed independently by 2 reviewers. Discrepancies were resolved through discussion or by consulting a third reviewer. For full texts without access, authors were contacted to request the file to be made available. All randomized controlled trials (RCTs) and quasi-RCTs of app-supported pregnancy lifestyle interventions and related qualitative and quantitative research across the different study phases were considered for inclusion. GWG or GDM had to be a primary or secondary outcome in the studies to be included. Studies that began in the preconception period were eligible for inclusion if the intervention continued during pregnancy and if women were followed up throughout pregnancy to assess GWG and GDM outcomes.

### Stage 4: Charting of the Data

A template for data charting was developed by the review team and pretested. Data charting was conducted by 1 reviewer and verified by 2 other reviewers. Discrepancies were discussed, and a final consensus document was produced. Study characteristics, such as author, year, recruitment status, and country, as well as PICOS characteristics and findings on GWG and GDM were extracted from the RCTs. Information on the types of evidence and app characteristics, functionalities, and tracking devices was gathered for all studies, including those with published reports of app development but without available information on the planned RCT.

### Stage 5: Collating, Summarizing, and Reporting of the Results

The study selection process is described narratively and presented in a PRISMA (Preferred Reporting Items for Systematic Reviews and Meta-Analyses) flow diagram [52]. Extracted data from the included studies were categorized in the most meaningful ways, coded, synthesized by basic descriptive analysis, and presented in tabular and graphical formats. All findings are also summarized narratively.

## Results

### Study Selection and Sources of Evidence

**Figure 1:**
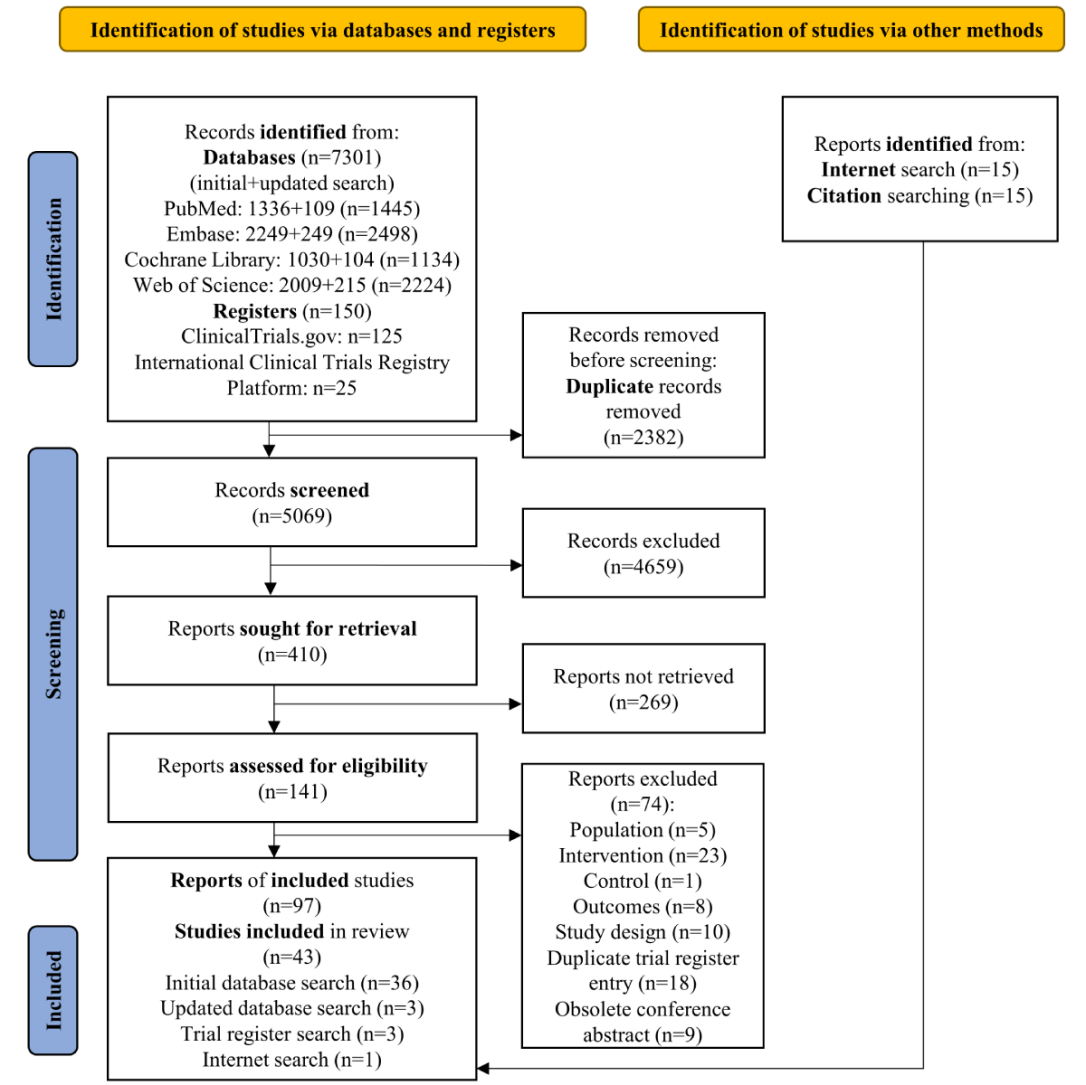
PRISMA (Preferred Reporting Items for Systematic Reviews and Meta-Analyses) 2020 flow diagram showing the identification and selection of studies.

Figure 1 depicts the flow of identified and included studies in this review. A total of 7301 records were identified from the initial and updated database searches. Trial register searches yielded an additional 150 records. After removing duplicates, 68.03% (5069/7451) of the records were screened by title. Of these 5069 records, 4659 (91.91%) were discarded, resulting in 410 (8.09%) remaining reports that were sought for retrieval by screening the abstract according to the PCC criteria. Of these 410 reports, although 269 (65.6%) were not retrieved, 141 (34.4%) were assessed in full for eligibility. On the basis of the PICOS and PCC criteria, of these 141 reports, 74 (52.5%) were excluded at this stage. Finally, 67 (69%) reports from the database and trial register searches together with 15 (15%) eligible reports identified via internet search and 15 (15%) eligible reports identified by citation searching, amounting to a total of 97 reports from 43 studies, were included in this scoping review. Of the 97 included reports, 38 (39%) were TREs [53-90], 49 (50%) were full peer-reviewed articles [25,26,38-42,91-132], 5 (5%) were conference abstracts [133-137], 2 (2%) were preprints [138,139], 1 (1%) was a master’s thesis [140], 1 (1%) was a dissertation [141], and 1 (1%) was a grant report [142].

### Scope of the Literature

**Figure 2:**
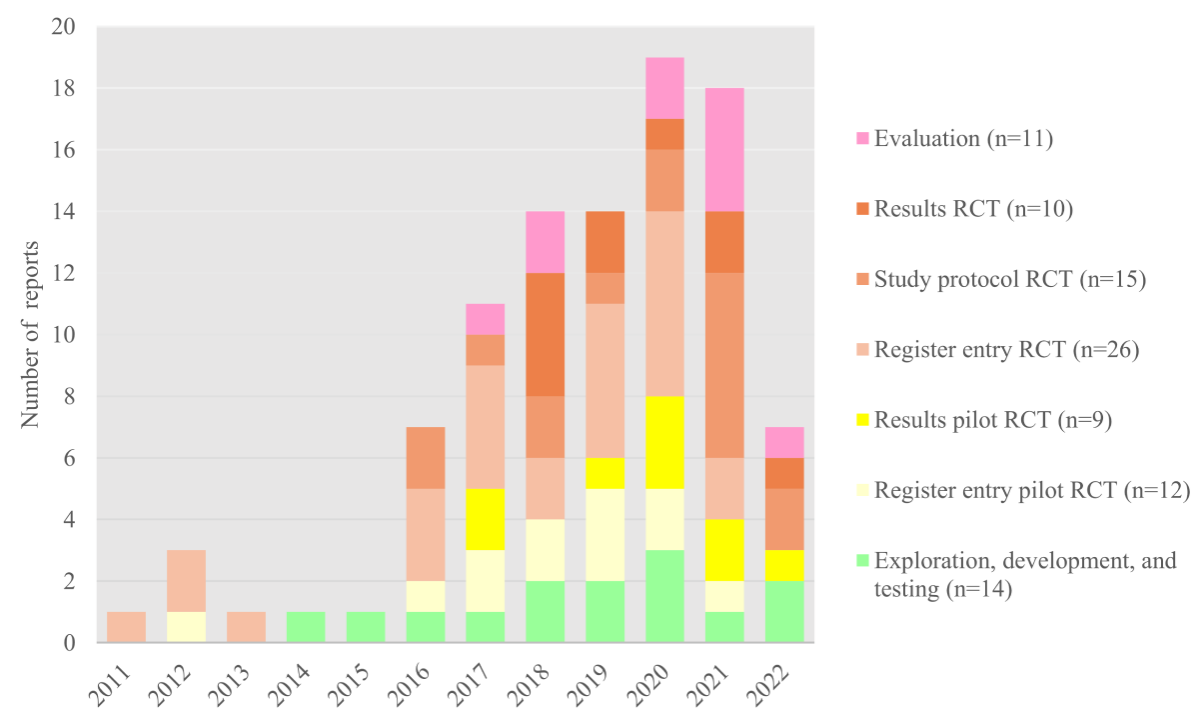
Number and type of identified reports per year up to June 2022 (a total of 97 reports were included). RCT: randomized controlled trial.

Figure 2 presents the scope of the available literature up to June 2022 in absolute numbers of published reports. All 97 reports were included in this overview. In 2011, the first eHealth app–supported RCT was registered [62]. The first mHealth app–supported RCTs were registered in 2012 [78,80,87]. The first article describing the development of an eHealth app–supported lifestyle intervention was published in 2014 [100]. One year later, the first publication on an intervention involving the development of an mHealth app followed [40]. In 2016, study protocols of effectiveness RCTs with app support were published for the first time [117,123]. The results of pilot RCTs with app support and a report evaluating an app-supported intervention were first available in 2017 [98,127,140]. The results of efficacy or effectiveness RCTs were first reported in 2018 [39,99,118,141]. Overall, the number of newly published reports steadily increased from 2015 onward: of the 97 included reports, 1 (1%) was published in 2015, up to 19 (20%) were published in 2020, and 18 (19%) were published in 2021. Overall, TREs accounted for the largest proportion of published reports (38/97, 39%). Multimedia Appendix 3 [25,26,38-42,53-142] lists the reports by study and intervention phase.

### PICOS Characteristics of Included RCTs and Findings on GWG and GDM Outcomes

#### Overview

Table 2 provides an overview of the PICOS characteristics of all identified RCTs in this review (39/43 studies, 91%). Details on study and intervention characteristics are presented in Multimedia Appendix 4 [38,39,41,42,53-91,93-95,97,99-111,​^113^-^115^,^117^,^118^,^120^-^127^,^129^,^130^,^134^-^136^,^138^-^141^]. Descriptive analyses are based on the coded data that are presented in Multimedia Appendix 5 [25,26,38-42,53-142]. Studies planning to conduct an RCT in the future (4/43, 9%) were not considered in this overview because no information on study characteristics was available [116,119,128,131].

**Table 2:** Population, intervention, control, outcomes, and study design (PICOS) characteristics of the included randomized controlled trials (RCTs) with available information (n=39).

Characteristic and category			RCTs, n (%)
**Study design**			
	Pilot	13 (33)	
	Efficacy or effectiveness	26 (67)	
**Study arms**			
	2	34 (87)	
	3	5 (13)	
**Sample size**			
	≤100	14 (36)	
	>100-1000	17 (44)	
	>1000	8 (21)	
**Recruitment status**			
	Recruiting	14 (36)	
	Completed	17 (44)	
	Active, not recruiting	2 (5)	
	Unknown	4 (10)	
	Withdrawn or terminated	2 (5)	
**Recruitment timing**			
	Preconception	3 (8)	
	First trimester	9 (23)	
	Until or within second trimester	22 (56)	
	Until or within third trimester	3 (8)	
	Unknown	2 (5)	
**Country or continent**			
	United States	16 (41)	
	Europe	9 (23)	
	Asia	7 (18)	
	Australia	4 (10)	
	Canada	2 (5)	
	Multiple	1 (3)	
**Population**			
	All BMI categories	10 (26)	
	Normal weight + overweight + obesity	6 (15)	
	Overweight + obesity	16 (41)	
	Obesity	3 (8)	
	High risk for GDM^a^	4 (10)	
**Behavior change technique or framework**			
	Any	26 (67)	
	None	13 (33)	
**Implementation science framework**			
	Any	4 (10)	
	None	35 (90)	
**Intervention duration**			
	Preconception (+ pregnancy)	3 (8)	
	Pregnancy only	23 (59)	
	Pregnancy + post partum	10 (26)	
	Pregnancy only (intervention 1) and pregnancy + post partum (intervention 2)	1 (3)	
	Uncertain	2 (5)	
**Intervention mode of delivery**			
	App only	8 (21)	
	App + technology	15 (39)	
	App + nontechnology	6 (15)	
	App + technology + nontechnology	10 (26)	
**Lifestyle content of intervention**			
	Diet only	5 (13)	
	PA^b^ only	6 (15)	
	Only well-being or sleep or both	1 (3)	
	Diet + PA	15 (39)	
	Diet + PA + well-being or sleep or both	11 (28)	
	Uncertain	1 (3)	
**Intervention tailoring^c^**			
	Feedback based on tracked lifestyle data and goals	33 (85)	
	Individual coaching sessions	21 (54)	
	Adaptive according to risk	9 (23)	
**Control group**			
	Standard care	19 (49)	
	Oral or written information	8 (21)	
	App or website alternative	8 (21)	
	Tracking device	4 (10)	
**Results on GWG^d^ and GDM outcomes**			
	Any results reported	18 (46)	
	No results reported	21 (54)	

^a^GDM: gestational diabetes mellitus.

^b^PA: physical activity.

^c^Independent of the mode of delivery.

^d^GWG: gestational weight gain.

#### Study Characteristics

Of the included RCTs, 44% (17/39) had been completed, and 36% (14/39) were still recruiting. Whereas 67% (26/39) of the RCTs investigated efficacy or effectiveness, 33% (13/39) were pilot studies investigating the feasibility of interventions. Most of the studies (34/39, 87%) were 2-armed, whereas 5 (13%) studies were 3-armed, comparing 2 intervention groups and 1 control group. Of these 5 studies, 2 investigated 2 different physical activity programs [55,102], whereas 1 compared 2 different intervention lengths (pregnancy vs pregnancy and post partum) [99], 1 compared an app group with an app + coaching group [41], and 1 compared 2 different delivery modes (in person vs app) [127]. The sample size of all RCTs ranged from 26 [42] to 2039 [77] participants, with 44% (17/39) of the studies recruiting between 100 and 1000 participants (Table 2). More than half of the studies (22/39, 56%) recruited women until or within the second trimester. Of the 39 studies, 9 (23%) limited the recruitment of women to the first trimester and 3 (8%) to the preconception period, whereas 3 (8%) included women until or within the third trimester. Studies were conducted in the United States (16/39, 41%), Europe (9/39, 23%), Asia (7/39, 18%), Australia (4/39, 10%), and Canada (2/39, 5%), whereas 1 (3%) of the 39 studies was conducted across multiple countries [95].

#### Population

Most of the studies included women with overweight and obesity (16/39, 41%) and from all BMI categories (10/39, 26%). Fewer studies excluded women with underweight (6/39, 15%), focused on women with a high risk for GDM (4/39, 10%), or only included those with obesity (3/39, 8%).

#### Intervention

In 59% (23/39) of the RCTs, the intervention was delivered in pregnancy only, whereas 26% (10/39) continued the intervention post partum, and 3% (1/39) assessed 2 time frames. Of the 39 RCTs, 3 (8%) started the intervention before conception, whereas in 2 (5%), the time frame was uncertain. The intervention involved an app combined with further technological components, including tracking devices or telephone calls, in 39% (15/39) of the RCTs. In 8 (21%) of the 39 studies, an app was the only intervention component used. An app and nontechnological intervention components, such as face-to-face visits or leaflets, were applied in 6 (15%) of the 39 studies, whereas in 10 (26%), the intervention was complemented by both technological and nontechnological components. More than one-third of the studies (15/39, 39%) addressed diet and physical activity within their interventions, and 11 (28%) of the 39 studies additionally involved well-being or sleep or both. Fewer studies focused only on diet (5/39, 13%) or physical activity (6/39, 15%). Of the 39 studies, 1 (3%) focused only on well-being and sleep. Independent of the mode of delivery, the interventions of included studies were tailored to a certain degree. Most of the studies (33/39, 85%) provided individual feedback based on tracked lifestyle data and goals. Of the 39 studies, 21 (54%) offered individual coaching sessions to participants; in 9 (23%), the intervention was adaptive in response to the individual risk profile. Of the 39 RCTs, 26 (67%) based the intervention on behavior change frameworks or techniques, such as motivational interviewing (n=8); specific, measurable, achievable, reasonable, and time-bound (SMART) goals (n=8); or social cognitive theory (n=7). Of the 39 RCTs, 4 (10%) applied implementation science frameworks, including intervention mapping; the exploration, preparation, implementation, and sustainment (EPIS) framework; and the reach, effectiveness, adoption, implementation, and maintenance (RE-AIM) framework [94,95,102,107].

#### Control

Control groups received standard care only in nearly half of the studies (19/39, 49%). In some of the studies, the control group had access to an alternative app or website (8/39, 21%) or tracking device (4/39, 10%). Additional oral or written information was delivered to control groups in 8 (21%) of the 39 studies.

#### Outcomes

Of the 39 included RCTs, 37 (95%) assessed GWG, and 24 (62%) assessed GDM, as either primary or secondary outcomes. Of the 39 RCTs, 18 (46%) had published results on GWG outcomes. Of these 18 RCTs, 8 (44%) were pilot RCTs [41,42,105,109,125,127,139,140], and 10 (56%) were efficacy or effectiveness RCTs [39,99,103,111,115,118,120,​^134^,^138^,^141^]. Significant intervention effects on any GWG outcome were found in 7 (39%) of these 18 studies [39,103,115,118,120,127,134], whereas 11 (61%) observed no effect. Of the 39 RCTs, 5 (13%) reported GDM results, but none observed a significant intervention effect [39,103,115,118,120].

#### App Characteristics

All 43 included studies provided some information on the apps they used. Multimedia Appendix 6 [25,26,38-42,53-142] provides a detailed overview of app characteristics and functionalities as well as tracking devices used in the studies. Descriptive analyses were based on coded data that are presented in Multimedia Appendix 5.

Most of the studies (30/43, 70%) used an mHealth app, 16% (7/43) used an eHealth app, and 9% (4/43) used a hybrid format or applied both an eHealth and an mHealth app. In 2 (5%) of the 43 studies, the type of app was uncertain. In more than half of the studies (24/43, 56%), research teams either developed new apps or adjusted the content of existing systems, 30% (13/43) used commercially available apps, and 5% (2/43) used both. For 9% (4/43) of the studies, no information on app origin was available.

The tracking and self-monitoring of health and lifestyle data were possible in the apps in 88% (38/43) of the studies; most often, it was possible to track weight, physical activity, diet, or a combination of these. Most of the studies (31/43, 72%) provided educational material via the app using different media types, such as text, video, and audio. Goal setting was possible via the app in 19 (44%) of the 43 studies. Feedback to participants via the app was provided in 58% (25/43) of the studies, whereas 51% (22/43) used prompts within the apps as reminders, feedback, motivation, or to inform about health and lifestyle data being outside of normal ranges. Communication with peers (eg, through social networks) via the app was possible in 7 (16%) of the 43 studies. Gamification elements, including quizzes and rewards, were integrated into the app in 6 (14%) of the 43 studies. Health coaching via the app was delivered in 5 (12%) of the 43 studies. Health and lifestyle data were tracked using wearable or Bluetooth-connected devices in approximately half of the studies (21/43, 49%). Most often, an activity tracker (15/43, 35%) or a weight scale (12/43, 28%) was used. Of the 43 studies, 1 (2%) used a blood pressure cuff.

## Discussion

### Principal Findings

After more than 3 decades of research on conventional, primarily face-to-face lifestyle interventions in pregnancy, a new era of digital interventions is currently emerging with apps showing promising potential to overcome previous barriers to implementation and dissemination. This scoping review identified 97 reports of 43 planned, ongoing, and completed studies on app-supported lifestyle interventions during pregnancy to manage GWG and prevent GDM. The steady increase in published reports from 2015 onward, most of which were TREs (38/97, 39%), and approximately one-third of the identified RCTs were still recruiting (14/39, 36%), indicates that this nascent field is developing. The studies were found to have published reports across different study phases involving exploration, intervention planning, app development and testing, and implementation and evaluation covering qualitative and quantitative research. The studies differed in their characteristics and intervention approaches, posing challenges in identifying the most effective components and implementable approaches and underlining the importance of standardized, comprehensive intervention and outcome reporting.

Previous systematic reviews and meta-analyses on technology-supported lifestyle interventions identified only a small number of app-based studies and observed mixed findings while being unable to draw firm conclusions owing to the limited available evidence from mostly pilot RCTs [43-47].

In this review, we included 39 app-supported RCTs, of which 8 pilot and 10 efficacy or effectiveness trials (18/39, 46%) provided results on GWG outcomes. In 7 of these 18 (39%) trials, the rates of excessive GWG or total GWG could be reduced by the intervention. Of the 39 RCTs, 5 (13%) efficacy or effectiveness trials reported results on GDM and observed no effect, which is in line with previous concerns on the overall effectiveness of lifestyle interventions on clinical outcomes and requires further elucidation [143]. On the basis of the limited number of published results on GWG and GDM outcomes (18/39, 46% of RCTs), it is currently unclear as to whether and to what extent app-supported pregnancy lifestyle interventions are effective and suitable for broad application at the population level. Moreover, the complexity and heterogeneity of intervention approaches complicate the differentiation between more or less effective components to ascertain which are the most effective. A planned systematic review and meta-analysis with a rigorous risk-of-bias assessment will provide a more comprehensive and profound interpretation of findings.

The included studies varied in their design, population, intervention, and app characteristics. The studies were mostly conducted in high-income countries and focused on high BMI categories as an inclusion criterion. Populations considered socioeconomically disadvantaged as well as minority populations should be given more consideration in future studies because they are often the most vulnerable to adverse health outcomes in pregnancy [144,145]. Furthermore, a greater diversity of women at risk could be reached in interventions if risk factors other than high BMI would be considered in studies (eg, by using validated screening tools). One of the included studies applied the validated Monash GDM screening tool to identify women at high risk for GDM [95]. A validated screening tool to identify women at high risk for excessive GWG has recently been developed by our group [146]. Such scores could be integrated into apps and applied in clinical health care settings.

Intervention timing and duration differed among the studies. A large proportion of the studies delivered the intervention during pregnancy, with fewer studies continuing the intervention after pregnancy and only 3 studies starting the intervention before conception. The best timing and duration for intervening are still uncertain. However, the importance of intervening early and the need for more holistic approaches spanning from preconception to the postpartum period have recently been emphasized [2,14,147,148].

Intervention content that was delivered in the included studies primarily focused on healthy nutrition and physical activity, whereas fewer studies addressed mental health and well-being aspects in their programs. Perinatal anxiety and depression are common and have been linked to adverse behavioral and health outcomes in women [149,150]. Thus, mental health and well-being should be given more attention in interventions, as also suggested by the Health in Preconception, Pregnancy and Postpartum Global Alliance [2]. Within apps, provided content should be presented in a concise, easy way, with focus on visualization and the integration of various delivery forms, including text, audio, and video formats [26]. Moreover, women appreciate content that is tailored to their individual preferences, culture, and lifestyles as well as the consideration of social surroundings, pregnancy symptoms, and previous knowledge and risks [26,113,116].

Individualization is a key determinant for intervention success because women differ in their needs and barriers regarding lifestyle support. Previously, tailored advice was often given in individual coaching sessions, requiring time and resources. Apps and connected devices enable readily available lifestyle advice and the continuous monitoring of health and lifestyle data with individual goal setting and feedback on progress. These functionalities and BCTs have been applied in most of the included studies and could potentially help to increase motivation and engagement. However, women might get annoyed if they receive prompts at unfavorable times or too frequently [112]. In addition, although some women value the opportunity to self-monitor health behaviors and receive feedback on their progress, tracking can be perceived as time consuming, and some women might feel extra pressure when goals are not reached, prompting negative emotions [40-42,112,116]. Body weight is a particularly sensitive topic on which women would value sensitive support [97]. Thus, sensitive handling and the opportunity for women to individually adapt functionalities according to their own preferences are suggested to ensure continued app engagement. None of the studies used artificial intelligence–based tools, such as chatbots. Such internet-based assistance and self-learning tools could facilitate the timely delivery of personalized advice and thus increase intervention efficiency. The adaptation of interventions according to risk profiles based on monitored data is another promising strategy for improving precision prevention and the potential efficiency of interventions while saving time, costs, and resources [32]. In the future, maternal phenotyping could be improved by collecting data on clinical and mental health parameters, such as glucose and blood pressure levels, mood, and sleep. Moreover, newly emerging artificial intelligence–based diet assessment tools could also be used to facilitate the tracking of nutritional behavior and nutrient intake.

Overall, lifestyle interventions are increasing in complexity, and it will be a future challenge to differentiate the effects of intervention components and identify the most effective and implementable approaches to overcome the existing translational research gap. Grounding intervention development, implementation, and evaluation on implementation science frameworks could aid in the successful implementation of interventions [151]. Among the studies included in this review, only a few made use of such frameworks. However, all studies can contribute to translating research findings into practice by reporting on contextual, implementation, and process evaluation outcomes. Moreover, facilitators and barriers at the patient, health care provider, and system levels should be examined and considered.

### Strengths and Limitations

The review has several strengths and limitations. It is based on relevant frameworks and the searching and inclusion of reports was conducted without language and publication status restrictions. Register entries and gray literature sources were included to provide a comprehensive overview of the ongoing literature. However, by including these evidence sources, the often limited and not always up-to-date information available from these reports made the identification and classification of key characteristics challenging. In the case of an included ongoing RCT with limited information, it was uncertain whether the study accurately met the predetermined inclusion criteria because the intervention may have been conducted only in the preconceptional phase and not continued during pregnancy, but an assessment of GWG and GDM outcomes is planned [54]. Given the exploratory and comprehensive nature of this review and the importance of early intervention, we decided to include the study [54]. We considered qualitative and quantitative investigations, whereby no systematic approach to qualitative research synthesis was used. This scoping review did not consider reviews and meta-analyses for inclusion. The studies used different terminologies and often lacked clear definitions with regard to apps and digital devices, which made classification and interpretation often difficult. As this is a scoping review, quality assessment has not been conducted, which precluded a profound interpretation of the findings on GWG and GDM outcomes in the included RCTs.

### Implications for Future Research

This review has identified research gaps and provided suggestions for future app and intervention development. The increasing number of identified studies in the field justifies the conduct of systematic reviews and meta-analyses, summarizing qualitative and quantitative data across all study phases. To increase comparability and enable useful synthesis, ongoing and future studies are encouraged to use current guidelines, frameworks, and BCT taxonomies to guide outcome reporting [152-156]. It will be exciting to see which of the apps developed in research settings make it to market and to assess their impact as well as evaluate their quality using tools such as the Mobile App Rating Scale [157].

### Conclusions

This scoping review provides, for the first time, a comprehensive overview of ongoing research in the field of app-supported lifestyle interventions to manage GWG and prevent GDM. Although various approaches are emerging, it remains to be determined whether the interventions are effective and which app features and elements are the most beneficial. It also needs to be elucidated whether these interventions can be implemented outside of trial settings. This has been missed to be considered by most of the previous research in this area. As the entire process from app development to the implementation of an app-supported intervention into practice requires time and resources, it may be reasonable for future investigations to collaborate more intensively across disciplines and build upon previous research and available resources.

## Appendix

Multimedia Appendix 1 PRISMA-ScR (Preferred Reporting Items for Systematic Reviews and Meta-Analyses extension for Scoping Reviews) checklist.

Multimedia Appendix 2 Full electronic search strategies applied to databases and clinical trial registers.

Multimedia Appendix 3 Categorization of published reports by study and type of evidence.

Multimedia Appendix 4 Population, intervention, control, outcomes, and study design (PICOS) characteristics of the included randomized controlled trials.

Multimedia Appendix 5 Coding of extracted data.

Multimedia Appendix 6 Characteristics of the apps and tracking devices used in the 43 included studies.

## Abbreviations

BCT: behavior change technique
EPIS: exploration, preparation, implementation, and sustainment
GDM: gestational diabetes mellitus
GWG: gestational weight gain
mHealth: mobile health
PCC: population, concept, and context
PICOS: population, intervention, control, outcomes, and study design
PRISMA: Preferred Reporting Items for Systematic Reviews and Meta-Analyses
PRISMA-ScR: Preferred Reporting Items for Systematic Reviews and Meta-Analyses extension for Scoping Reviews
RCT: randomized controlled trial
RE-AIM: reach, effectiveness, adoption, implementation, and maintenance
SMART: specific, measurable, achievable, reasonable, and time-bound
TRE: trial register entry

## Data Availability

Data obtained or analyzed in this review are included in this document and its multimedia appendices.
