# Supplementary material for: App-Supported Lifestyle Interventions in Pregnancy to Manage Gestational Weight Gain and Prevent Gestational Diabetes: Scoping Review
Source: J Med Internet Res. 2023 Nov 10;25:e48853. doi: 10.2196/48853 (PMC10674147; doi:10.2196/48853)
Supplement: Multimedia Appendix 2 [file jmir_v25i1e48853_app2.docx]

Multimedia Appendix 2: Full electronic search strategies applied to databases and clinical trial registers.

| # | Search string**^a^** | Search hits |
| --- | --- | --- |
| **PubMed** | | |
| #1 | ("mhealth"[Title/Abstract] OR "m-health"[Title/Abstract] OR "mobile"[Title/Abstract] OR "app"[Title/Abstract] OR "application*"[Title/Abstract] OR "technolog*"[Title/Abstract] OR "ehealth"[Title/Abstract] OR "e-health"[Title/Abstract] OR "*phone*"[Title/Abstract] OR "Cell Phone"[MeSH Terms] OR "mobile applications"[MeSH Terms] OR "telemedicine"[MeSH Terms]) | 1,874,016 |
| #2 | ("pregnan*"[Title/Abstract] OR "gestation*"[Title/Abstract] OR "prenatal"[Title/Abstract] OR "pre-natal"[Title/Abstract] OR "antenatal"[Title/Abstract] OR "ante-natal"[Title/Abstract] OR "preconception*"[Title/Abstract] OR "pre conception*"[Title/Abstract] OR "pregnancy"[MeSH Terms] OR "pregnant women"[MeSH Terms]) | 1,132,767 |
| #3 | ("diet*"[Title/Abstract] OR "lifestyle"[Title/Abstract] OR "life-style"[Title/Abstract] OR "physical activity"[Title/Abstract] OR "exercis*"[Title/Abstract] OR "nutrition*"[Title/Abstract] OR "behavio*"[Title/Abstract] OR "health*"[Title/Abstract] OR "exercise"[MeSH Terms] OR "diet"[MeSH Terms] OR "health behavior"[MeSH Terms] OR "Life style"[MeSH Terms] OR "Behavior Therapy"[MeSH Terms] OR "Nutrition Therapy"[MeSH Terms] OR "prenatal care"[MeSH Terms]) | 5,355,363 |
| #4 | ("weight"[Title/Abstract] OR "gestational diabetes"[Title/Abstract] OR "gdm"[Title/Abstract] OR "gwg"[Title/Abstract] OR "weight gain"[MeSH Terms] OR "diabetes, gestational"[MeSH Terms]) | 889,935 |
| #5 | ("animals"[MeSH Terms] NOT "humans"[MeSH Terms]) | 4,829,176 |
| #6 | (((("mhealth"[Title/Abstract] OR "m-health"[Title/Abstract] OR "mobile"[Title/Abstract] OR "app"[Title/Abstract] OR "application*"[Title/Abstract] OR "technolog*"[Title/Abstract] OR "ehealth"[Title/Abstract] OR "e-health"[Title/Abstract] OR "*phone*"[Title/Abstract] OR "Cell Phone"[MeSH Terms] OR "mobile applications"[MeSH Terms] OR "telemedicine"[MeSH Terms])) AND (("pregnan*"[Title/Abstract] OR "gestation*"[Title/Abstract] OR "prenatal"[Title/Abstract] OR "pre-natal"[Title/Abstract] OR "antenatal"[Title/Abstract] OR "ante-natal"[Title/Abstract] OR "preconception*"[Title/Abstract] OR "pre conception*"[Title/Abstract] OR "pregnancy"[MeSH Terms] OR "pregnant women"[MeSH Terms]))) AND (("diet*"[Title/Abstract] OR "lifestyle"[Title/Abstract] OR "life-style"[Title/Abstract] OR "physical activity"[Title/Abstract] OR "exercis*"[Title/Abstract] OR "nutrition*"[Title/Abstract] OR "behavio*"[Title/Abstract] OR "health*"[Title/Abstract] OR "exercise"[MeSH Terms] OR "diet"[MeSH Terms] OR "health behavior"[MeSH Terms] OR "Life style"[MeSH Terms] OR "Behavior Therapy"[MeSH Terms] OR "Nutrition Therapy"[MeSH Terms] OR "prenatal care"[MeSH Terms]))) AND (("weight"[Title/Abstract] OR "gestational diabetes"[Title/Abstract] OR "gdm"[Title/Abstract] OR "gwg"[Title/Abstract] OR "weight gain"[MeSH Terms] OR "diabetes, gestational"[MeSH Terms])) | 1,469 |
| #7 | ((((("mhealth"[Title/Abstract] OR "m-health"[Title/Abstract] OR "mobile"[Title/Abstract] OR "app"[Title/Abstract] OR "application*"[Title/Abstract] OR "technolog*"[Title/Abstract] OR "ehealth"[Title/Abstract] OR "e-health"[Title/Abstract] OR "*phone*"[Title/Abstract] OR "Cell Phone"[MeSH Terms] OR "mobile applications"[MeSH Terms] OR "telemedicine"[MeSH Terms])) AND (("pregnan*"[Title/Abstract] OR "gestation*"[Title/Abstract] OR "prenatal"[Title/Abstract] OR "pre-natal"[Title/Abstract] OR "antenatal"[Title/Abstract] OR "ante-natal"[Title/Abstract] OR "preconception*"[Title/Abstract] OR "pre conception*"[Title/Abstract] OR "pregnancy"[MeSH Terms] OR "pregnant women"[MeSH Terms]))) AND (("diet*"[Title/Abstract] OR "lifestyle"[Title/Abstract] OR "life-style"[Title/Abstract] OR "physical activity"[Title/Abstract] OR "exercis*"[Title/Abstract] OR "nutrition*"[Title/Abstract] OR "behavio*"[Title/Abstract] OR "health*"[Title/Abstract] OR "exercise"[MeSH Terms] OR "diet"[MeSH Terms] OR "health behavior"[MeSH Terms] OR "Life style"[MeSH Terms] OR "Behavior Therapy"[MeSH Terms] OR "Nutrition Therapy"[MeSH Terms] OR "prenatal care"[MeSH Terms]))) AND (("weight"[Title/Abstract] OR "gestational diabetes"[Title/Abstract] OR "gdm"[Title/Abstract] OR "gwg"[Title/Abstract] OR "weight gain"[MeSH Terms] OR "diabetes, gestational"[MeSH Terms]))) NOT (("animals"[MeSH Terms] NOT "humans"[MeSH Terms])) | 1,336 |
| **Web of Science** | | |
| #1 | TS=( mhealth or "m-health" or mobile or app or application* or technolog* or ehealth or "e-health" or *phone*) AND TS=( pregnan* or gestation* or prenatal or "pre-natal" or antenatal or "ante-natal" or preconception* or "pre conception") AND TS=( diet* OR lifestyle OR "life-style" OR "physical activity" OR exercis* OR nutrition* OR behavio* OR health*) AND TS=(weight OR "gestational diabetes" OR gdm OR gwg) | 2,076 |
| #2 | NOT (TI = animal* OR AK = animal* OR KP= animal* OR SU= veterinary sciences) NOT (TI = human* OR AK = human* OR KP= human*) | 911,932 |
| #3 | #1 NOT #2 | 2,009 |
| **Cochrane Library** | | |
| #1 | (mhealth or "m-health" or mobile or app or application* or technolog* or ehealth or "e-health" or phone or smartphone):ti,ab,kw | 117794 |
| #2 | (pregnan* or gestation* or prenatal or "pre-natal" or antenatal or "ante-natal" or preconception* or "pre-conception"):ti,ab,kw | 78450 |
| #3 | (diet* OR lifestyle OR "life-style" OR "physical activity" OR exercis* OR nutrition* OR behavio* OR health*):ti,ab,kw | 564617 |
| #4 | (weight OR "gestational diabetes" OR gdm OR gwg):ti,ab,kw | 116779 |
| #5 | #1 AND #2 AND #3 AND #4 | 1002 |
| #6 | MeSH descriptor: [Pregnancy] explode all trees | 22464 |
| #7 | MeSH descriptor: [Pregnant Women] explode all trees | 300 |
| #8 | MeSH descriptor: [Telemedicine] explode all trees | 2764 |
| #9 | MeSH descriptor: [Cell Phone] explode all trees | 1,815 |
| #10 | MeSH descriptor: [Mobile Applications] explode all trees | 765 |
| #11 | MeSH descriptor: [Exercise] explode all trees | 25,356 |
| #12 | MeSH descriptor: [Life Style] explode all trees | 5,620 |
| #13 | MeSH descriptor: [Health Behavior] explode all trees | 36,403 |
| #14 | MeSH descriptor: [Diet] explode all trees | 18,804 |
| #15 | MeSH descriptor: [Nutrition Therapy] explode all trees | 9,561 |
| #16 | MeSH descriptor: [Behavior Therapy] explode all trees | 17,152 |
| #17 | MeSH descriptor: [Prenatal Care] explode all trees | 1,534 |
| #18 | MeSH descriptor: [Weight Gain] explode all trees | 2,616 |
| #19 | MeSH descriptor: [Diabetes, Gestational] explode all trees | 988 |
| #20 | #1 OR #8 OR #9 OR #10 | 119,190 |
| #21 | #2 OR #6 OR #7 | 78,672 |
| #22 | #3 OR #11 OR #12 OR #13 OR #14 OR #15 OR #16 OR #17 | 582,753 |
| #23 | #4 OR #18 OR #19 | 116,844 |
| #24 | #20 AND #21 AND #22 AND #23 | 1,030 |
| **Embase** | | |
| #1 | (pregnan* or gestation* or prenatal or "pre-natal" or antenatal or "ante-natal" or preconception* or "pre conception").ti,ab,kw. | 790,196 |
| #2 | (diet* or lifestyle or "life-style" or "physical activity" or exercis* or nutrition* or behavio* or health*).ti,ab,kw. | 5,862,238 |
| #3 | (weight or "gestational diabetes" or gdm or gwg).ti,ab,kw. | 1,069,140 |
| #4 | exp mobile phone/ or exp mobile application/ or exp telemedicine/ | 88,958 |
| #5 | exp pregnancy/ or exp pregnant woman/ | 576,213 |
| #6 | exp exercise/ or exp diet/ or exp diet therapy/ or exp lifestyle/ or exp health behavior/ or exp behavior therapy/ or exp prenatal care/ | 1,523,937 |
| #7 | exp body weight gain/ or exp pregnancy diabetes mellitus/ | 60,364 |
| #8 | (exp animal/ or nonhuman/) not exp human/ | 5,413,590 |
| #9 | (mhealth or "m-health" or mobile or app or application* or technolog* or ehealth or "e-health" or phone* or smartphone*).ti,ab,kw. | 2,138,391 |
| #10 | 4 or 9 | 2,174,648 |
| #11 | 1 or 5 | 925,731 |
| #12 | 2 or 6 | 6,396,332 |
| #13 | 3 or 7 | 1,085,608 |
| #14 | 10 and 11 and 12 and 13 | 2,414 |
| #15 | 14 not 8 | 2,249 |

^a^ Searches were conducted on May 17, 2021 and November 11, 2021

**Search terms applied to ClinicalTrials.Gov and the International Clinical Trial Registry Platform ^a^**

**Intervention:** mhealth OR "m-health" OR mobile OR app OR application OR ehealth OR "e-health"

**Condition or disease:** weight OR "gestational diabetes" OR gdm OR gwg OR diet OR dietary OR lifestyle OR "life-style" OR "physical activity" OR exercise OR nutrition OR nutritional OR behavior OR behaviour

**Title:** pregnant OR pregnancy OR gestation OR prenatal OR "pre-natal" OR antenatal OR "ante-natal" OR preconception OR "pre-conception"

^a^ Searches were conducted on May 30, 2022
