# Supplementary material for: App-Supported Lifestyle Interventions in Pregnancy to Manage Gestational Weight Gain and Prevent Gestational Diabetes: Scoping Review
Source: J Med Internet Res. 2023 Nov 10;25:e48853. doi: 10.2196/48853 (PMC10674147; doi:10.2196/48853)
Supplement: Multimedia Appendix 3 [file jmir_v25i1e48853_app3.docx]

Multimedia Appendix 3: Categorization of published reports by study and type of evidence.

| **Study Acronyms** | **Exploration/**  **development/testing** | **Register entry pilot RCT** | **Results pilot RCT** | **Register entry RCT** | **Study protocol RCT** | **Results RCT** | **Evaluation** |
| --- | --- | --- | --- | --- | --- | --- | --- |
| BabyScripts | [93] |  |  | [53] |  | [91,134] | [92,133,135] |
| Begin Better | [25, 94] |  |  | [54] |  |  |  |
| BlossomUP |  | [55] | [140] |  |  |  |  |
| Bump2Baby and Me |  |  |  | [56] | [95] |  |  |
| BumptUp |  | [57] |  |  |  |  |  |
| BurnAlong |  | [58] |  |  |  |  |  |
| DIGITAL-G |  |  |  | [59] |  |  |  |
| Eating4Two | [40] |  |  | [60] | [96] |  | [97] |
| E-HEALTH |  |  |  | [61] |  |  |  |
| e-Moms Roc | [100] |  |  | [62] |  | [99] | [98] |
| ePPOP-ID |  |  |  | [63] | [101] |  |  |
| Fit MUM |  | [64] | [41] |  |  |  |  |
| FitMum |  |  |  | [65] | [102] |  |  |
| GeMuKi |  |  |  | [66] | [104] | [103] |  |
| GlycoLeap |  |  | [105] |  |  |  |  |
| GROWell |  |  |  | [67] | [106] |  |  |
| H42/H4U Pilot |  | [69] | [42] |  |  |  |  |
| H42/H4U Effectiveness | [132] |  |  | [68] | [107] |  |  |
| Health Empowerment Program |  |  |  | [70] |  | [138] |  |
| Healthy for my Baby |  |  |  | [71] | [108] |  |  |
| Healthy Mom2B |  |  |  | [72] |  | [141] |  |
| Healthy Mom Zone |  | [73] | [109] |  | [110] |  |  |
| HealthyMoms | [26] |  |  | [74] | [38] | [111] | [112] |
| HHIPBe | [137] | [75] |  |  |  |  |  |
| INTER-ACT | [113] |  |  | [76] | [114] |  |  |
| Kaiser Permanente |  |  |  | [77] |  |  |  |
| LGI Diet |  |  |  | [78] |  | [115] |  |
| mHELP | [116] |  |  |  |  |  |  |
| Mobile Medical Platform |  |  |  | [79] |  |  |  |
| MOMFIT |  |  |  | [80] | [117] | [118] |  |
| MyHealthyPregnancy | [119] |  |  |  |  |  |  |
| NEAT!2 |  | [81] |  |  |  |  |  |
| PaMPPr |  | [82] |  |  |  |  |  |
| Pas & Pes |  |  |  | [83] |  | [120] |  |
| Pears |  |  |  | [84] | [123] | [39] | [121,122,136] |
| PLAN |  | [85] | [125] |  |  |  | [124] |
| PurUmeed Aaghaz |  |  |  | [86] | [126] |  |  |
| Smart Moms |  |  |  | [87] |  | [127] |  |
| Smart Moms Canada | [128] |  |  |  |  |  |  |
| Smart Moms in WIC/ Healthy Beginnings |  |  |  | [88] | [129] |  |  |
| SpringMom |  |  |  | [89] | [130] |  |  |
| StartSmart | [131, 142] |  |  |  |  |  |  |
| STRIDE |  | [90] | [139] |  |  |  |  |

Abbreviations: RCT, randomized controlled trial
