# Supplementary material for: App-Supported Lifestyle Interventions in Pregnancy to Manage Gestational Weight Gain and Prevent Gestational Diabetes: Scoping Review
Source: J Med Internet Res. 2023 Nov 10;25:e48853. doi: 10.2196/48853 (PMC10674147; doi:10.2196/48853)
Supplement: Multimedia Appendix 4 [file jmir_v25i1e48853_app4.docx]

Multimedia Appendix 4: Population, intervention, control, outcomes, and study design (PICOS) characteristics of the included randomized controlled trials.

| **Study name/acronym, author(s), references** | **Study design, recruitment status, country** | **Sample size (n)** | **Population characteristics** | **Intervention characteristics** | **Control characteristics** | **Results GWG/GDM outcomes** |
| --- | --- | --- | --- | --- | --- | --- |
| **BabyScripts**  Marko et al., 2019; Drexler et al., 2020 (conference abstract); DeNicola et al., 2018; Marko et al., 2016; TRE 2016 (NCT02914301)  [53,91,93,134,135] | Design: Multicentre, effectiveness, two-arm, quasi-RCT (historical control)  Status: Completed Country: USA | Marko et al. 2021: 118  (I: 60, C: 58); TRE: 181; Drexler et al. 2020, conference abstract: 368 (I: 182,  C: 186) | BMI ≤ 38 kg/m^2^ (Drexler et al., 2020, conference abstract) "Low risk" | Recruitment: ≤ 13 GW Duration: Pregnancy Technological components: Baby Scripts mobile app + connected BP cuff and weight scale Non-Technological components: NA Content: General pregnancy info, nutrition, GWG (non-personalized)  Tailoring: Feedback, adaptive BCT/Framework: NA | Standard prenatal care (historical control group) | GWG (S) (Drexler et al., 2020, conference abstract)  N analyzed: I: 182, C: 186 Total GWG (Mean (SD)) (kg): I: 13.2 (4.2), C: 14.8 (4.2), *p* < 0.001 Excessive total GWG (n (%)): I: 69/182 (37.9%), C: 94/186 (50.8%) Adequate total GWG (n (%)): I: 69/182 (37.9%), C: 63/186 (34.1%) Inadequate total GWG (n (%)): I: 44/182 (24.2%), C: 28/186 (15.1%) GWG according to IOM: *p* = 0.02 |
| **Begin Better**  Scott et al., 2022; TRE 2021 (ACTRN12621000128897)  [54,94] | Design: Two-arm effectiveness RCT  Status: Recruiting Country: Australia | Target (TRE): 864 (parallel assignment); Scott et al. 2022: 870 | BMI ≥ 25 kg/m^2^ | Recruitment: Preconception Duration: Preconception Technological components: Begin Better web app Non-Technological components: One consultation at trial entry, fortnightly individual health coaching sessions Content: General preconception care infos, nutrition, PA, GWG, mental health  Tailoring: Feedback, coaching BCT Framework: Psychological theories (acceptance and commitment therapy, cognitive behavioural therapy), information-motivation-behavioural skills model, BCTs  Implementation Science Framework: Intervention Mapping | Consultation with midwife at trial entry; standard preconception care advice within the Begin Better web app | GWG (S), GDM (S) |
| **BlossomUp**  TRE 2016 (NCT02909725); Master's Thesis McKinney 2017  [55,140] | Design: Three-arm (pilot) RCT,  Status: Unknown (Status was: Recruiting) Country: USA | Estimated enrolment (TRE): 36 Actual enrolment: 11  (I1: 5, I2: 3,  C: 3) | BMI < 40 kg/m^2^, inactive women | Recruitment: 16–22 GW Duration: Pregnancy Technological components: Fitbit mobile app + connected device  (I1: Fitbit Alta as self-monitoring behaviour change tool to decrease sedentary time, I2: FitBit Charge to record goal of accumulating 150 minutes of moderate-vigorous PA/week)  Non-Technological components: NA Content: PA  Tailoring: Feedback BCT/Framework: NA | Continue normal daily routine | GDM (P), GWG (S)  N analyzed: I1: 2, I2: 3, C: 3 Excessive total GWG (n (%)): I1: 1/2 (50%), I2: 3/3 (100%), C: 2/3 (75%) |
| **Bump2Baby and Me**  O'Reilly et al., 2021; TRE 2020 (ACTRN12620001240932)  [56,95] | Design: Multicentre two-arm implementation and effectiveness RCT Status: Recruiting Countries: Australia, Ireland, UK, Spain | Aim: 800  (I: 400,  C: 400) | High risk of developing GDM | Recruitment: < 24 GW Duration: Pregnancy + postpartum Technological components: Bump2Baby & Me mobile coaching app + connected weight scale Non-Technological components: NA Content: Nutrition, PA, well-being, individualized, adaptive  Tailoring: Feedback, coaching, adaptive BCT/Framework: BCTs, Behaviour Change Wheel  Implementation Science Framework: EPIS, RE-AIM | Standard care, Bump2Baby & Me website, newsletters, reminders to visit website | GWG (S), GDM (S) |
| **BumptUp**  TRE 2020 (NCT04480931)  [57] | Design: Two-arm feasibility and potential efficacy pilot RCT  Status: Active, not recruiting Country: USA | Aim: 40 (parallel assignment) Actual enrolment: 38 | All BMI classes | Recruitment: 18–20 GW Duration: Pregnancy + postpartum Technological components: BumptUp mobile app Non-Technological components: NA Content: PA  Tailoring: NA BCT/Framework: NA | Evidence-based educational brochure about PA during pregnancy | GWG (S), GDM (S) |
| **BurnAlong**  TRE 2018 (NCT03551535)  [58] | Design: Two-arm feasibility and adherence RCT Status: Withdrawn (study IRB approval expired) Country: USA | 50  (I: 25, C: 25) | At risk for GDM (History of GDM, or BMI ≥ 30 kg/m^2^, or two or more first degree relatives with GDM) | Recruitment: Withdrawn Duration: Pregnancy Technological components: BurnAlong (hybrid) app, FitBit heart rate + activity monitors Non-Technological components: NA Content: PA  Tailoring: Feedback BCT/Framework: NA | Standard counselling regarding PA recommendations in pregnancy | GWG (S), GDM (S) |
| **DIGITAL-G**  TRE 2019 (NCT03987412)  [59] | Design: Two-arm (effectiveness) RCT Status: Unknown (Status was: Recruiting) Country: China | Estimated Enrolment: 1200 | One or more risk factors for GDM | Recruitment: ≤ 12 GW Duration: Pregnancy Technological components: Mobile app incorporating nutrition, exercise and psychological support Non-Technological components: Face-to-face education  Content: Nutrition, PA, mental health, individualized  Tailoring: Feedback, adaptive BCT/Framework: NA | Regular pregnancy care | GDM (P) |
| **Eating4Two**  Davis et al., 2018; TRE 2017 (ACTRN12617000169347)  [60,97] | Design: Two-arm effectiveness RCT Status: Recruiting Country: Australia | Aim: 1330  (1:1) | All BMI classes, varying levels of socioeconomic advantage and disadvantage | Recruitment: < 15 GW Duration: Pregnancy Technological components: Eating4Two mobile app  Non-Technological components: NA Content: Nutrition, GWG, adaptive (Discussion of GWG with maternity caregivers encouraged when deviation from recommended levels)  Tailoring: Feedback, adaptive BCT/Framework: Information-motivation-behavioural skills approach to behaviour change | Usual antenatal care, booklet “Good nutrition in pregnancy” | GWG (P), GDM (S) |
| **E-HEALTH**  TRE 2017 (NCT05094479)  [61] | Design: Two-arm (pilot) effectiveness RCT Status: Completed Country: Finland | Aim: 1000 (parallel assignment) Actual enrolment: 1047 | All BMI classes | Recruitment: < 28 GW Duration: Pregnancy Technological components: E-health/web app Non-Technological components: NA Content: Non-personalized information on healthy lifestyle habits (GWG, Nutrition, PA)  Tailoring: NA BCT/Framework: NA | Health app without health information | GWG (P) |
| **e-Moms Roc**  Graham et al., 2014; Olson et al., 2018; TRE 2011 (NCT01331564)  [62,99,100] | Design: Three-arm effectiveness RCT Status: Completed Country: USA | 1689 (I1: 563,  I2: 563,  C: 563) | BMI ≥ 18.5 and < 35 kg/m^2^ | Recruitment: ≤ 20 GW Duration: I1: Pregnancy I2: Pregnancy and postpartum Technological components: Interactive website (web app) Non-Technological components: NA Content: Nutrition, PA, GWG  Tailoring: Feedback BCT/Framework: Integrative Model of Behavioural Prediction, Behaviour Model for Persuasive Design, BCTs | Access to control website during pregnancy and postpartum - information tools (local resources, articles, FAQs), event reminders, blogging feature | GWG (P)  N analyzed: I1+I2: 1126, C: 563 Total GWG (Mean (SD)) (kg):  I1+I2: 13.73 (0.46), C: 13.73 (0.45) Adjusted estimate (95% CI): 0.10 (-0.58, 0.77), *p* = 0.78 Excessive total GWG (n (%)): I1+I2: 48.1% (SD = 2.0%),  C: 46.2% (SD = 2.4%) Adjusted estimate (95% CI):  1.09 (0.98, 1.20), *p* = 0.12 |
| **ePPOP-ID**  Deruelle et al., 2020; TRE 2016 (NCT02924636)  [63,101] | Design: Multicentre two-arm efficacy RCT Status: Recruiting Country: France | 860 (1:1) | BMI 30–40 kg/m^2^ | Recruitment: 12–22 GW (TRE: 12–19) Duration: Pregnancy and postpartum Technological components: Web-based platform by BePatient accessible through web or (mobile) app, reminder emails Non-Technological components: NA Content: Nutrition, PA, well-being, personalized  Tailoring: NA BCT/Framework: BCT | Standard care with oral information about the goal of nutritional needs during pregnancy and GWG guidelines according to BMI | GWG (S), GDM (S) |
| **Fit MUM**  Darvall et al., 2020; TRE 2017 (ACTRN12617000038392)  [41,64] | Design: Three-arm feasibility RCT Status: Completed Country: Australia | 30  (I1: 10,  I2: 10,  C:10) | BMI ≥ 30 kg/m^2^ | Recruitment: 12–16 GW Duration: Pregnancy Technological components: I1: App-group: pedometer synced to “Fitbit” app to self-monitor daily step counts I2: App-coach-group: additional behavioural change program by health coach including 3 phone sessions Non-Technological components: I2: One face-to-face session Content: PA, nutrition  Tailoring: Feedback, coaching  BCT/Framework: Self-determination theory, SMART goals | Pedometer not linked to smartphone (synced manually at clinic appointments) | GWG (S) N analyzed: I1: 9, I2: 10, C: 8  (Dropout rate: 10%)  Total GWG (Mean (SD)) (kg): I1: 7.91 (4.17), I2: 13.21 (5.73), C: 13.22 (5.91) |
| **FitMum**  Roland et al., 2021; TRE 2018 (NCT03679130)  [65,102] | Design: Single-site, three-arm efficacy RCT Status: Active, not recruiting Country: Denmark | 220 (I1: 88,  I2: 88,  C: 44) | BMI  18.5–45 kg/m^2^ | Recruitment: ≤ 15 GW Duration: Pregnancy Technological components: “Garmin connect” app, Garmin activity tracker, weekly SMS reminders Non-Technological components:  I1 (EXE): Weekly structured supervised exercise training I2 (MOT): Four individual and three group counselling sessions  Content: PA  Tailoring: Feedback, coaching BCT/Framework: Motivational interviewing, self-determination theory and BCTs  Implementation Science Framework/Design: RE-AIM | Standard care and activity tracker that only shows time and battery life | GWG (S), GDM (S) |
| **GeMuKi**  Nawabi et al., 2020; Krebs et al., 2022; TRE 2019 (DRKS00013173)  [66,103,104] | Design: Two-arm effectiveness-implementation RCT Status: Recruitment completed, follow-up still ongoing Country: Germany | Aim: 1860 Recruited: 1466  (I: 792,  C: 674) | All BMI classes | Recruitment: < 12 GW Duration: Pregnancy and postpartum Technological components: GeMuKi app Non-Technological components: Up to six counselling sessions of about 10 min each Content: Nutrition, PA, breastfeeding, substance use  Tailoring: Feedback, coaching BCT/Framework: Motivational interviewing, SMART goals | Standard care | GWG (P), GDM (S)  N analyzed: C: 636, I: 744 Total GWG (Mean (kg)):  C: 14.2, I: 13.3, MD (95%CI):  -0.97 (-1.56 to -0.38), *p* = 0.001 Excessive total GWG (%): C: 59.6% I: 52.8%, Adjusted OR  (95% CI): 0.76 (0.60 to 0.96), *p* = 0.024 Incidence of GDM: I: 12.4%, C: 11.3%, Adjusted OR  (95% CI): 1.12 (0.77 to 1.63), *p* = 0.537 |
| **GlycoLeap**  Li et al., 2019  [105] | Design: Two-arm pilot feasibility, acceptability and preliminary utility RCT Status: NA/ Completed (no TRE) Country: Singapore | 30  (I: 15, C: 15) | BMI ≥ 25 kg/m^2^ | Recruitment: 18–20 GW Duration: Pregnancy Technological components: Glyoleap food coaching mobile app  Non-Technological components: NA Content: Nutrition  Tailoring: Feedback BCT Framework: NA | Standard dietary orientation, information on recommended GWG, simple PA pointers | GWG (P)  N analyzed: C: 14, I: 12 8-week FU (26-28 GW):  Total GWG (Mean (SD)) (kg): I: 2.9 (1.9), C: 3.0 (2.24), *p* = 0.92,  MD (β (95% CI): -0.08 (-1.80 to 1.63),  *p* = 0.92 Adequate total GWG (n (%)): I: 67% (8/12), C: 36% (5/14) |
| **GROWell**  Simmons et al., 2022; TRE 2020 (NCT04449432)  [67,106] | Design: Two-arm efficacy RCT Status: Recruiting Country: USA | 480  (I: 240,  C: 240) | BMI  25–< 40 kg/m^2^ | Recruitment: 10–16 GW Duration: Pregnancy and postpartum Technological components: GROWell mobile app, Bluetooth scale Non-Technological components: NA Content: Nutrition, personalized  Tailoring: Feedback BCT/Framework: Self-regulation theory | Attention control, text messaging on pregnancy information (without dietary information) | GWG (P), GDM (S) |
| **H42/H4U Pilot**  Coughlin et al., 2020; TRE 2018 (NCT03551054)  [42,69] | Design: Two-arm pilot feasibility and acceptability RCT Status: Completed Country: USA | 26  (I: 13, C: 13) | BMI  ≥ 18.5 kg/m^2^ | Recruitment: ≤ 12 GW (TRE),  Coughlin et al. 2020: 11–16 GW Duration: Pregnancy and postpartum Technological components: Lose It! App (manual data entry), coach calls (providing feedback on tracking information), electronic learning materials Non-Technological components: Paper based learning materials Content: Nutrition, PA, GWG, wellbeing  Tailoring: Feedback, coaching BCT/Framework: (COACH model), motivational interviewing, BCTs | Standard care, educational materials on basic nutrition and safety information, no self-monitoring recommendations, no individualized GWG counselling | GWG (P)  N analyzed: C: 13, I: 13 Total GWG (Mean (SD)) (kg): I: 11.4 (4.5), C: 12.0 (4.5) Adequate total GWG (% (n)): I: 77% (10/13), C: 54% (7/13), *p* = 0.41 |
| **H42/H4U Effectiveness**  Bennett et al., 2022; TRE 2021 (NCT04724330)  [68,107] | Design: Two-arm effectiveness and implementation RCT Status: Recruiting Country: USA | Aim: 380 (1:1) | BMI  ≥ 25.0 kg/m^2^ | Recruitment: ≤ 15 GW Duration: Pregnancy and postpartum Technological components: Interactive web-based platform connected to digital scale, Fitbit mobile app, health coach phone contacts Non-Technological components: NA Content: Nutrition, PA, GWG, well-being  Tailoring: Feedback, coaching, adaptive BCT/Framework: Motivational interviewing, COACH model, BCTs, SMART goals  Implementation Science Framework: PRECIS-2-PS | Standard care (typical, evidence- and guideline-based experience in prenatal care clinics) | GWG (P), GDM (S) |
| **Health Empowerment Program**  Chen et al., 2021 (preprint); TRE 2020 (NCT04553731)  [70,138] | Design: Two-phase two-arm app development and efficacy/effectiveness RCT Status: Completed Country: Taiwan | 92  (I:46, C:46) | BMI  ≥ 25 kg/m^2^  (in TRE: > 25 kg/m^2^) | Recruitment: 8–12 GW (TRE), Chen et al. 2020: < 17 GW Duration: Pregnancy Technological components: MyHealthyWeight mobile app, wearable activity tracker (WAT (Mi Band 5)), SMS Non-Technological components: NA Content: PA, nutrition, GWG  Tailoring: Feedback BCT/Framework: Social-cognitive theory, BCTs | Standard antenatal care | GWG (P)  N analyzed: First trimester (I: 46, C: 46), Second trimester (I: 40, C: 43), Third trimester (I: 37, C: 43)  Mean dropout rate: 13.04% Total GWG (Mean (SD)) (kg): First trimester: I: 0.70 (2.90), C: 1.24 (2.75), *p* = 0.260 Second trimester: I: 1.82 (3.98), C: 3.11 (3.47), *p* = 0.117 Third trimester: I: 6.34 (5.66), C: 7.43 (4.99), *p* = 0.365 Excessive total GWG (n (%)): I: 8/37 (21.6%), C: 14/43 (32.6%),  *p* = 0.275 |
| **Healthy for My Baby**  Hardy et al., 2021; TRE 2020 (NCT04242069)  [71,108] | Design: Two-phase, two-arm multicentre open parallel group effectiveness RCT Status: Recruiting  Country: Canada | Estimated enrolment (TRE): 68 (1:1) Partner involvement! | BMI  ≥ 25.0 kg/m^2^ | Recruitment: Intend to conceive within 12 months of trial enrolment Duration: Preconception and pregnancy Technological components: Mobile app Non-Technological components: Two MI counselling sessions with the partner Content: Nutrition, PA, sleep, well-being, environment (tobacco use etc.)  Tailoring: Feedback, coaching, adaptive BCT/Framework: Control Theory, Motivational interviewing, SMART goals | Standard care in preconception period and pregnancy | GWG (S), GDM (S) |
| **Healthy Mom2B**  Dahl, 2018 (Dissertation);  TRE 2017 (NCT03063528)  [72,141] | Design: Two-arm effectiveness RCT Status: Completed Country: USA | TRE (actual enrolment): 142;  Dahl, 2018: 140  (I: 77, C: 63) | BMI ≥ 18.5 kg/m^2^, predominantly white (81.6%) and highly educated (58.6% beyond Bachelor's degree | Recruitment: ≤ 20 GW Duration: Pregnancy Technological components: MakeMe mobile app for facilitating group-based behaviour challenges, HM2B website (web app) for tracking health outcomes (e.g., GWG) and receiving info, email reminders, podcasts Non-Technological components: NA Content: Nutrition, PA, (GWG)  Tailoring: Feedback BCT/Framework: Unified Theory of Acceptance and Use of Technology, which draws on Social Cognitive Theory and the Theory of Planned Behaviour | MakeMe^TM^ Mobile App, non-diet/PA challenges (i.e., stress reduction and stress management), relaxation and meditation podcasts | GWG (P)  N analyzed: I: 47, C: 40 Total GWG (Mean (SE)) (kg): I: 11.37 (0.74), C: 10.43 (0.80), *p* = 0.39 Excessive total GWG (% (n)): I: 42.6% (20/47), C: 52.5% (21/40),  *p* = 0.42 Adequate total GWG (% (n)):  I: 36.2% (17/47), C: 25.0% (10/40) Inadequate total GWG (% (n)):  I: 21.3% (10/47), C: 22.5% (9/40) |
| **Healthy Mom Zone**  Downs et al., 2018; Downs et al., 2021; TRE 2019 (NCT03945266)  [73,109,110] | Design: Two-arm feasibility RCT Status: Completed Country: USA | 31 (I: 15, C: 16) | BMI 25–45 kg/m^2^  (TRE different: 24 – > 40 kg/m^2^) | Recruitment: > 8 – ≤ 12 GW (TRE: 6–16) Duration: Pregnancy Technological components: MyFitnessPal mobile app for evaluation of diet quality, Wi-Fi weight scale, activity monitor, self-monitoring after goal setting/action planning  Non-Technological components: Weekly face-to-face counselling sessions, 60 min each Content: GWG, Nutrition, PA, info on foetal growth, adaptive (step up dosages)  Tailoring: Feedback, coaching, adaptive BCT/Framework: Theory of planned behaviour, BCTs, self-regulation | Standard care | GWG (P)  N analyzed: I: 13, C: 14  Dropout rate: 13% Post-Pre weight (GWG) (kg) (Mean change (95% CI)): I: 6.9 (3.8, 10.0), C: 8.8 (5.1, 12.3),  MD (95% CI): -1.9 (-6.6 to 2.9), *p* = 0.43 |
| **HealthyMoms**  Henriksson et al., 2019; Sandborg et al., 2021; TRE 2017 (NCT03298555)  [38,74,111] | Design: Two-arm parallel effectiveness RCT Status: Completed Country: Sweden | 300  (I: 150,  C: 150) | All BMI classes | Recruitment: First trimester Duration: Pregnancy Technological components: HealthyMoms mobile app Non-Technological components: NA Content: Nutrition, PA, GWG  Tailoring: Feedback BCT/Framework: Social-cognitive theory, BCTs | Standard antenatal care (included an optional lecture in early pregnancy on a healthy lifestyle) | GWG (P)  N analyzed: I: 134, C: 137 Excessive total GWG (n (%)): I: 67/134 (50.0%), C: 68/137 (49.6%) Adequate total GWG (n (%)): I: 52/134 (38.8%), C: 48/137 (35.0%) Inadequate total GWG (n (%)): I: 15/134 (11.2%), C: 21/137 (15.3%) |
| **HHIPBe**  TRE 2020 (NCT04336878)  [75] | Design: Two-arm feasibility and acceptability RCT Status: Recruiting Country: UK | 80  (I: 40, C: 40) | BMI ≥ 25.0 kg/m^2^ and < 38.0 kg/m^2^ | Recruitment: NA (early pregnancy) Duration: Pregnancy and postpartum Technological components: Mobile app to self-monitor weight and behaviours Non-Technological components: Brief 1:1 session to encourage the development of ten healthy habits, self-guided leaflet, record keeping logbook Content: Nutrition, PA, GWG  Tailoring: Coaching BCT/Framework: NA | Standard antenatal care | GWG (P) |
| **INTER-ACT**  Bogaerts et al., 2017; Bogaerts et al., 2020; TRE 2017 (NCT02989142)  [76,113,114] | Design: Multicentre two-arm effectiveness RCT Status: Unknown (Status was: Recruiting) Country: Belgium | 1100 (I: 550,  C: 550) | Excessive GWG in previous pregnancy | Recruitment: Day 2 or 3 postpartum Duration: Postpartum/preconception and pregnancy Technological components: INTER-ACT mobile app connected to scale and pedometer, motivational email reminders Non-Technological components: Face-to-face counselling Content: Nutrition, PA, well-being, GWG  Tailoring: Feedback, coaching BCT/Framework: Social cognitive theory, SMART goals, BCTs | Standard care | GDM (P), GWG (S) |
| **Kaiser Permanente**  TRE 2019 (NCT03880461)  [77] | Design: Two-arm effectiveness cluster-RCT Status: Recruiting Country: USA | 2040 (1:1) | BMI 25 - < 40 kg/m^2^ | Recruitment: < 15 GW Duration: NA Technological components: Hybrid app, Phone counselling session(s), text messages, emails, mhealth tools Non-Technological components: NA Content: Lifestyle, GWG, personalized, adaptive  Tailoring: Feedback, coaching, adaptive BCT/Framework: Motivational interviewing | Standard antenatal care | GWG (P) |
| **LGI Diet**  Zhang et al., 2019; TRE 2012 (NCT01628835)  [78,115] | Design: Two-arm effectiveness RCT Status: Completed Country: China | 400  (I: 200,  C: 200) | BMI ≥ 24 kg/m^2^ | Recruitment: ≤ 16 GW Duration: Pregnancy Technological components: Mobile app DietGI, phone interviews Non-Technological components: 3 diet consultation interviews, individualized diet plan, diet GI and glycemic load calculations Content: Nutrition, individualized  Tailoring: Feedback, coaching BCT/Framework: NA | Standard nutrition and PA consultation, advice on GWG, individualized diet plan | GWG (P), GDM (P)  N analyzed: I: 200, C: 200 (imputed data) Total GWG (Mean (SD)) (kg): I: 9.6 (7.4), C: 11.2 (6.3), *p* = 0.02 (extracted from Table 2, in the text  *p* = 0.03 is written) Incidence of GDM (n (%)): I: 45 (22.5%), C: 43 (21.5%), *p* = 0.33 |
| **Mobile Medical Platform**  TRE 2021 (NCT04989634)  [79] | Design: Two-arm effectiveness cluster-RCT  Status: Recruiting Country: China | 2000  (I: 1000,  C: 1000) | All BMI classes | Recruitment: 8–12 GW Duration: Pregnancy Technological components: APP software Non-Technological components: Face-to-face teaching Content: Nutrition, PA, weight, individualized  Tailoring: Coaching BCT/Framework: NA | Standard antenatal care | GDM (P), GWG (S) |
| **MOMFIT**  LIFE-Moms Research Group, 2016; van Horn et al., 2018; TRE 2012 (NCT01631747)  [80,117,118] | Design: Two-arm (effectiveness) RCT Status: Completed Country: USA | TRE & LIFE-Moms Research Group, 2016: 300; Van Horn et al., 2018:  281 (I: 140, C: 141) | BMI 25 - < 40 kg/m^2^ | Recruitment: < 16 GW Duration: Pregnancy Technological components: LOSEIT! app to track food intake, MOMFIT website, weekly e-mails, coaching calls (MI), individual and group counselling sessions via phone/webinar, log activity, text messages, electronic handouts Content: Nutrition, PA, sleep, individualized  Tailoring: Feedback, coaching BCT/Framework: Motivational interviewing | Standard care, MOMFIT website, publicly available guidelines and recommendations on PA, nutrition, heathy pregnancy | GWG (P), GDM (S)  N analyzed: I: 140, C: 140 Total GWG (Mean (SD)) (kg): I: 10 (6), C: 12 (6), *p* (adjusted) = 0.02 Excessive total GWG (n (%)): I: 96 (68.6), C: 119 (84.4),  *p* (adjusted) = 0.004 Incidence of GDM (%): I: 5.3%, C: 7.1%, *p* (adjusted) = 0.41 |
| **NEAT!2**  TRE 2021 (NCT04903574)  [81] | Design: Two-arm pilot effectiveness and perception RCT  Status: Recruiting Country: USA | 28 (I: 14,  C: 14) | > 18.5 kg/m^2^ NW, OW, OB | Recruitment: < 16 GW Duration: Pregnancy Technological components: NEAT!2 sedentary behaviour mobile app Non-Technological components: NA Content: PA  Tailoring: Feedback BCT/Framework: NA | Commercially available pregnancy mobile/smart-phone app | GWG (P), GDM (P) |
| **PaMPPr**  TRE (NCT03802734)  [82] | Design: Two-arm pilot feasibility and acceptability RCT Status: Unknown (Status was: Enrolling by invitation) Country: Canada | 128 (1:1) | All BMI classes | Recruitment: 20–30 GW Duration: Uncertain Technological components: Mindful meditation mobile app, actigraph to measure sleep Non-Technological components: Sleep info leaflet Content: Mindfulness mediation, sleep  Tailoring: NA BCT/Framework: NA | General pregnancy sleep leaflet, actigraph to measure sleep | GDM (S) |
| **Pas & Pes**  Gonzalez-Plaza et al., 2022; TRE 2018 (NCT03706872)  [83,120] | Design: Parallel two-arm effectiveness RCT Status: Terminated (Women's confinement for COVID-19 pandemic and changes on prenatal care) Country: Spain | 150 (I: 78, C:72) | BMI ≥ 30 kg/m^2^ | Recruitment: 12–18 GW Duration: Pregnancy Technological components: Smartband (Mi Band 2) and Mi Fit app to track PA and set goals, hangouts app for receiving health counselling and support Non-Technological components: NA Content: PA, GWG  Tailoring: Feedback, coaching BCT/Framework: Social cognitive theory | Oral information and written support material, Nutrition/PA/GWG advice for sedentary/inactive women or women with OW/OB | GWG (P), GDM (S)  N analyzed: I: 60, C: 53 Total GWG (Mean (SD)) (kg): I: 7.6 (5.5), C: 10.1 (6.4) MD (95% CI): 2.5 (0.2 to 4.7), *p* = 0.02 Excessive total GWG (n (%)): I: 21/60 (35%), C: 29/53 (55%),  *p* = 0.08 Adequate total GWG (n (%)): I: 21/60 (35%), C: 15/53 (28%) Inadequate total GWG (n (%)): I: 18/60 (30%), C: 9/53 (17%) Incidence of GDM (n (%)): I: 10 (15%), C: 12 (22%), *p* = 0.36 |
| **Pears**  Kennelly et al., 2016; Kennelly et al., 2018; Ainscough et al., 2018; O’ Sullivan 2020; Greene et al., 2021; TRE 2013 (ISRCTN29316280)  [39,84,121-123,136] | Design: Two-arm single centre effectiveness RCT Status: Completed Country: Ireland | 565  (I: 278, C:287) | BMI  25.0–39.9 kg/m^2^ | Recruitment: 10–15 GW Duration: Pregnancy Technological components: “healthy lifestyle package” including a mobile app, emails Non-Technological components: single 75 min educational session (low GI diet, PA advice), 2 FU visits Content: Nutrition, PA  Tailoring: Feedback, coaching BCT/Framework: Control Theory, SMART goals, Social Cognitive Theory, Com-B Model/Behaviour Change Wheel, BCTs | Usual antenatal care | GDM (P), GWG (S)  N analyzed: I: 278, C: 287 Total GWG (Mean (SD)) (Baseline to term) (kg): I: 11.3 (5.6), C: 12.6 (5.6) MD (95% CI): -1.3 (-2.49 to -0.15), *p* = 0.027 (unadjusted), *p* = 0.13 (adjusted)  Excessive total GWG (%):  I: 50.9%, C: 63.8%, *p* = 0.01 Incidence of GDM (n (%)): I: 37/241 (15.4%), C: 36/257 (14.1%) RR: 1.1, 95% CI 0.71-1.66, *p* = 0.71) |
| **PLAN**  Huang et al., 2019; Willcox et al., 2020; TRE 2017 (ACTRN12617000725369)  [85,124,125] | Design: Two-arm pilot feasibility and indication for potential efficacy RCT Status: Completed Country: Australia | 57  (I: 30, C: 27) | BMI ≥ 20 kg/m^2^ | Recruitment: < 11 GW Duration: Pregnancy Technological components: Web app, PLAN project website, SMS Non-Technological components: 1:1 session Content: Diet, PA, well-being  Tailoring: Feedback, coaching BCT/Framework: BCTs, SMART goals | Standard antenatal care | GWG (P), GDM (S) (Glucose tolerance by oGTT)  N analyzed (total GWG): I: 17, C: 17  N analyzed (GWG IOM): I: 23, C: 19  Total GWG (Mean (95% CI)) (kg):  I: 13.33 (11.63-15.03), C: 11.81 (9.80-13.82), *p* = 0.230  Excessive total GWG (n (%)): I: 13/23 (56.52%), C: 11/19 (57.89%) Adequate total GWG (n (%)): I: 5/23 (21.74%), C: 2/19 (10.53%) Inadequate total GWG (n (%)): I: 5/23 (21.74%), C: 6/19 (31.58%) |
| **PurUmeed Aaghaz**  Nuruddin et al., 2021; TRE 2020 (NCT04216446)  [86,126] | Design: Two-arm efficacy RCT Status: Recruiting Country: Pakistan | 300 (1:1) | All BMI classes | Recruitment: First trimester Duration: Pregnancy Technological components: Mobile app PurUmeed Aaghaz Non-Technological components: NA Content: Diet, PA, supplement use, personalized  Tailoring: Feedback BCT/Framework: Theory of planned behaviour, theory of self-efficacy, Fogg behaviour model, transtheoretical model for behaviour change | Face-to-face counselling, educational brochure on diet and PA, paper based food record diary | GWG (S), GDM (S) |
| **Smart Moms**  Redman et al., 2017; TRE 2012 (NCT01610752)  [87,127] | Design: Three-arm pilot effectiveness RCT Status: Completed Country: USA | 54 (I1: 18,  I2: 19,  C: 17) | BMI 25.0–39.9 kg/m^2^ | Recruitment: First trimester Duration: Pregnancy Technological components: Both groups received pedometer and Wi-Fi scale, 18 lessons on lifestyle and behaviour modification (delivery mode differed) I2: Smart Moms mobile app connected with Wi-Fi scale and pedometer Non-Technological components: I1: In-person, paper-based tracking of steps, GWG monitoring Content: Nutrition, PA, GWG  Tailoring: Feedback, coaching BCT/Framework: BCT Frameworks, SMART goals | Standard antenatal care | GWG (P)  N analyzed (GWG IOM):  I1: 18, I2: 19, C: 13 Total GWG (LS mean (SE)) (kg):  I1: 8.0 (1.3), I2: 10.0 (1.3), C: 12.8 (1.5) Excessive total GWG (n (%)): I1: 10/18 (56%), I2: 11/19 (58%),  C: 11/13 (84.6%),  I1 vs. C: *p* = 0.03,  I2 vs. C: *p* = 0.04 |
| **Smart Moms in WIC/ Healthy Beginnings**  Flanagan et al., 2020; TRE 2019 (NCT04028843)  [88,129] | Design: Two-arm, two-phase effectiveness RCT Status: Recruiting Country: USA | Aim: 432 | BMI  18.5–40 kg/m^2^ | Recruitment: < 16 GW Duration: Pregnancy and postpartum Technological components: Healthy Beginnings mobile app synchronized with Fitbit Alta and scale, facebook group, emails, youtube videos  Non-Technological components: NA Content: GWG, PA, Nutrition  Tailoring: Feedback, adaptive BCT/Framework: BCTs | Standard WIC program | GWG (P) |
| **SpringMom**  Kawasaki et al., 2021; TRE 2020 (UMIN000041460)  [89,130] | Design: Two-arm multicentre effectiveness RCT Status: Enrolling by invitation Country: Japan | TRE: 270;  Kawasaki et al., 2021: 452  (I: 226,  C: 226) | BMI  ≥ 25.0 kg/m^2^ | Recruitment: < 30 GW Duration: Pregnancy and postpartum Technological components: Mobile app (Calo Mama Plus Ninsanpu Course®) with personalized advice based on data (weight, body composition, PA, sleep) measured with connected IoT devices and meal records and photographs recorded by the app Non-Technological components: NA Content: GWG, sleep, PA, diet  Tailoring: Feedback BCT/Framework: NA | Standard antenatal care, IoT devices have to be worn during data collection (start, 1, 6, 12, 24 months postpartum) | GWG (S), GDM (S) |
| **STRIDE**  Thomas, 2021 (preprint); TRE 2019 (NCT03936283)  [90,139] | Design: Two-arm pilot feasibility and acceptability RCT  Status: Completed Country: USA | 68  (I: 33, C: 35) | BMI  25–40 kg/m^2^ | Recruitment: < 12 GW Duration: Pregnancy Technological components: Phone calls (motivational interviewing), mobile health website accessible via smartphone or computer connected with activity tracker and scale, email or text messages Non-Technological components: One baseline visit, guidebook Content: PA, nutrition, GWG  Tailoring: Feedback, coaching BCT/Framework: Motivational interviewing, Social Cognitive Theory, Transtheoretical model, BCTs | Standard prenatal care | GWG (P)  N analyzed: I: 33, C: 35 Total GWG (Mean (SD)) (kg): I: 12.7 (3.8), C: 12.1 (4.1), adjusted MD (95% CI): 1.14 (-0.71 to 3.00) |

Abbreviations: App, application; BCT, behaviour change technique; BMI, body mass index; BP, blood pressure; C, control group; CI, confidence interval; COACH, commit, omit, add, communicate, honour your wellness; EPIS, Exploration, Preparation, Implementation, Sustainment; FAQ, frequently asked question; FU, follow up; GDM, gestational diabetes mellitus; GI, glycaemic index; GW, gestational week; GWG, gestational weight gain; I, intervention group; IOM, Institute of Medicine; IoT, Internet of Things; LS, least square; MD, mean difference; NA, not applicable; NW, normal weight; OB, obesity; oGTT, oral glucose tolerance test; OR, odds ratio; OW, overweight; P, primary outcome; PA, physical activity; PRECIS-2-PS, PRagmatic Explanatory Continuum Indicator Summary-2; RCT, randomized controlled trial; RE-AIM, Reach, Efficacy-Adoption, Implementation, Maintenance; RR, relative risk; S, secondary outcome; SD, standard deviation; SE, standard error; SMART, specific, measurable, achievable, relevant, time-bound; SMS, short message service; TRE, trial register entry; WIC, Women, Infants, and Children; Wi-Fi, wireless fidelity
