# Supplementary material for: App-Supported Lifestyle Interventions in Pregnancy to Manage Gestational Weight Gain and Prevent Gestational Diabetes: Scoping Review
Source: J Med Internet Res. 2023 Nov 10;25:e48853. doi: 10.2196/48853 (PMC10674147; doi:10.2196/48853)
Supplement: Multimedia Appendix 6 [file jmir_v25i1e48853_app6.docx]

Multimedia Appendix 6: Characteristics of the apps and tracking devices used in the 43 included studies.

| **Study acronym, references** | **App name, operating system (iOS , Android, Windows, etc.)** | **Developed/**  **adapted vs. commercial** | **Wearable/Bluetooth-connected tracking device** | **Communication** | **Provider involvement** | **BCT/features** |
| --- | --- | --- | --- | --- | --- | --- |
| **BabyScripts**  [53,91-93,133-135] | Baby Scripts smartphone/mobile app (iOs, Android, or Windows-based) | Content developed | Wi-Fi connected weight scale and sphygmomanometer to measure BP | N/A | Clinicians will receive regular BP and weight measurements, early warnings to providers about aberrant data points | Non-personalized educational content (general pregnancy info, risks, nutrition, breastfeeding, GWG) Self-Monitoring (weight, BP), goal setting, automated feedback about goals; alerts depending on readings (early warnings to patients about aberrant data points) |
| **Begin Better**  [25,54,94] | Begin Better web app | Will be developed | N/A | With peers - Buddy system (participants are paired with another women) | N/A | Educational content (general pregnancy info, nutrition, PA, mental health), goal setting (weight, health, social, mental wellbeing), self-monitoring of goals, feedback,  create and upload habit action plans, habit formation, social support through buddy system, alerts (reminders, motivation), video and podcasts, further BCTs |
| **BlossomUp**  [55,140] | Fitbit mobile app (iOs and Android) | Commercial | SR GROUP: FitBit Alta (activity monitor), WALK GROUP: Fitbit Charge | N/A | N/A | Self-Monitoring, goal setting (PA, walking group), alerts (reminders), feedback (vibration if goal not reached) |
| **Bump2Baby and Me**  [56,95] | Smartphone/mobile app | Content developed | Digital scales (Smart Body Fat Scale, RENP HO) | mHealth coaching (synchronous (live-video feature) and asynchronous), Social Network (messaging with other women) | Health coach dashboard (commercial platform) | Personalized educational content, Goal setting (lifestyle), self-monitoring (weight), social network (messaging with other women), Alerts (reminders, Motivation), 1:1 coaching sessions, text and video messaging between coach and participant |
| **BumptUp**  [57] | BumptUp mobile app (no info on operating system- website info: “will be running soon”) | Developed (see website) | N/A | support from experts and mothers | N/A | Educational content (evidence-based workout information and education), goal setting (exercise and health related goals), self-monitoring, exercise plan, symptom tracking, social support (community forum), prompts (reminders, push notifications) (see Website info) |
| **BurnAlong**  [58] | Burn Along (hybrid) app, FitBit | Commercial | FitBit (HR and activity monitors) | N/A | N/A | Physical activity program FitBit HR and activity monitors to track adherence rates to prescribed exercise regimen  Alerts/immediate exercise compliance feedback”  (see also Website info) |
| **DIGITAL-G**  [59] | Mobile app | N/A | N/A | N/A | Based on monitoring data investigators will individualize suggestions | Educational content (diet, PA, mental health), self-Monitoring of diet, PA, weight, feedback, adaptive |
| **Eating4Two**  [40,60,96,97] | Eating4Two mobile app (iOs and Android, originally developed in Android) | Developed | N/A | N/A | When above or below GWG guidelines prompt to discuss issue with maternity caregiver | Educational content, self-monitoring (weight), feedback, alerts/prompts (Motivation, Reminders, Education) |
| **E-HEALTH**  [61] | E-health/web app | N/A | N/A | N/A | N/A | Non-personalized educational content (GWG, diet, PA) |
| **e-Moms Roc**  [62,98-100] | Web app | Developed | N/A | Blog | N/A | Educational content (pregnancy-related tips), tracking/self-monitoring (weight, diet, PA), feedback, PA and diet goal setting, weight gain tracker, barriers and strategies, reward, action plan, prompts |
| **ePPOP-ID**  [63,101] | BePatient web-based e-health platform (web app) | Developed by BePatient (commercial) according to program requirements | N/A | Connect with other participants (social network) and with caregivers (instant message system, forum) | Professional's module (remote follow-up of patient activities) | Educational content (behaviour, nutrition, breastfeeding, recipes), exercise and yoga videos, relaxation classes, self-monitoring of weight, prompts (motivation), social support through social network, quizzes (to evaluate the learning progress) |
| **Fit MUM**  [41,64] | Fitbit mobile app (iOs and Android) | Commercial | Fitbit Zip pedometer | N/A | N/A | Self-monitoring of PA |
| **FitMum**  [65,102] | Garmin Connect mobile app (iOs or Android) | Commercial | Garmin Vivosport (activity tracker, HR monitor, accelerometer) | N/A | N/A | Self-monitoring of PA |
| **GeMuKi**  [66,103,104] | GeMuKi mobile app | Developed | N/A | N/A | GeMuKi-Assist (counselling tool, access to data) | Educational content (health information on pregnancy), prompting SMART goals |
| **GlycoLeap**  [105] | Glycoleap smartphone/mobile app (iOs and google store platforms) | Commercial | N/A | Guidance from professional dietitians | N/A | Self-monitoring of diet (upload of food images), real-time detailed food coaching comments and guidance from dieticians (feedback) |
| **GROWell**  [67,106] | GROWell mobile app | Developed | Bluetooth scale | N/A | N/A | Self-monitoring of diet, personalized feedback, dietary goal setting, action planning, prompts (motivation, reminder, info) |
| **H42/H4U Pilot**  [42,69] | Lose It! mobile app | Commercial | N/A | N/A | Coaches used web-interface ("Ascend") to view tracking data | Self-monitoring of weight, diet, PA (manual entry) |
| **H42/H4U Effectiveness**  [68,107,132] | Interactive web-based platform (web app), Fitbit mobile app | Developed/adapted, commercial | home scale (EatSmart digital bathroom scale) | N/A | N/A | Educational content, tracking of weight via home scale, PA, nutrition, and weight tracking via Fitbit app |
| **Health Empowerment Program**  [70,138] | MyHealthyWeight mobile app (Android and iOs) | Developed | WAT (wrist worn Mi Band 5/wearable activity tracker) | N/A | N/A | Educational content, self-monitoring of PA, weight, diet (self-record), goal setting, reward, reminder, visual feedback |
| **Healthy for my Baby**  [71,108] | Mobile app | Developed | N/A | Platform to contact the research team | research team has access to information entered in mobile app - adaptation | Educational content, pregnancy related features (fertility calendar, research visit calendar), daily self-monitoring of lifestyle goals, goal setting, adaptation, prompts (reminders) |
| **Healthy Mom2B (HM2B)**  [72,141] | MakeMe mobile app, HM2B website Itunes and GooglePlay | Commercial (MakeMe App) + developed (HM2B Website) | N/A | Group-based challenges (communication between pregnant women) | Research team sets up weekly challenges, enters user info via back-end | Educational content, social support, reward, GWG tracking via website (plotted along IOM guidelines), podcasts, goal setting, group-based challenges (get points/gamification), self-monitoring, reminders via E-mail |
| **Healthy Mom Zone**  [73,109,110] | MyFitnessPal mobile app | Commercial | Wi-Fi weight scale, wrist worn PA tracker (“mhealth tools”) | N/A | N/A | Self-monitoring of dietary intake, weight, PA |
| **Healthy Moms**  [26,38,74,111,112] | Healthy Moms mobile app (Android and iOs) | Developed | N/A | N/A | N/A | Educational content, goal setting, self-monitoring of diet, PA, weight, tailored feedback, general pregnancy content, prompts |
| **HHIPBe**  [75,137] | N/A  (“access to an ‘app’ ”) | N/A | N/A | N/A | N/A | Self-monitor weight and behaviours (against 10 target behaviours), goal setting |
| **INTER-ACT**  [76,113,114] | Mobile app | Developed | Activity and sleep tracker (Withings GO) + Bluetooth connected weighing scale (Body Cardio Withings) | N/A | Coach website with dashboard (monitor results of self-weighing and daily steps) | Educational content, goal setting, self-Monitoring of diet, PA, weight, mood, visual feedback, prompts (motivation, reminders) |
| **Kaiser Permanente**  [77] | Hybrid, accessible via Smartphone or Website | Developed | “mHealth tool allowing data to be automatically transmitted to a mobile website” | N/A | N/A | Self-monitoring |
| **LGI Diet**  [78,115] | DietGI mobile app | Commercial | N/A | N/A | N/A | Meal tracking (selecting food types and amount of intake for every meal) -> Glycaemic index, Glycaemic load |
| **mHELP**  [116] | Web app | Developed | Can be synchronized with pedometer | N/A | N/A | Educational content, social support (Professional and Peer), self-monitoring, feedback, prompts (if above, below value), individualized goal setting |
| **Mobile Medical Platform**  [79] | N/A  (“APP software”) | N/A | N/A | N/A | N/A | Self-monitoring and evaluating diet, PA, weight |
| **MOMFIT**  [80,117,118] | LOSEIT! mobile app | Commercial | N/A | N/A | N/A | Track food intake, log PA in app |
| **MyHealthyPregnancy (MHP) App**  [119] | MHP mobile app | Developed | Digital weight scale | N/A | Real-time alerts to medical staff | Educational content, prompts (risk feedback and recommendations, reminders), self-monitoring of weight, individualized risk feedback, Voluntary daily "quizzes" to assess risk factors |
| **NEAT!2**  [81] | NEAT!2 sedentary behaviour mobile app | Commercial | ActivePAL (measure sedentary behaviour) | N/A | N/A | Prompts (reminders to stand up) |
| **PaMPPr**  [82] | Headspace mindful meditation mobile app | Commercial | Actigraph | N/A | N/A | Mindfulness meditation |
| **Pas & Pes**  [83,120] | Mi Fit mobile app, hangouts mobile app (Android and iOS) | Commercial | Smartband (Mi Band 2) linked to Mi Fit App | Contact with midwife through Hangouts app - women could ask questions that were solved with immediate response (< 1 hour) | N/A | Educational content and health counselling via Hangouts app - Goal setting of steps and weight via Mi Fit app, self-monitoring of weight, PA, social support, reward (prizes when goals achieved), prompts/vibration (prolonged inactivity, goal achievement) |
| **Pears**  [39,84,121-123,136] | Mobile app (Android and iOs) | Developed | N/A | N/A | N/A | Educational content, prompts (reminders) |
| **PLAN**  [85,124,125] | Web app | Developed | N/A | N/A | N/A | Self-monitoring, real-time feedback on GWG, Educational material, Goal setting, prompts (reminders, feedback)  (see also Website info) |
| **PurUmeed Aaghaz**  [86,126] | Online device dependent (mobile) app, Android and iOs | Developed | N/A | N/A | N/A | Educational content, self-monitoring of diet, PA, supplement use, individual advice, and feedback prompts (reminders) |
| **Smart Moms**  [87,127] | SmartMoms mobile app | Developed | Wireless bathroom scale; pedometer (Fitbit Zip) | N/A | N/A | Educational content, goal setting, self-monitoring of weight, diet, PA, data-driven feedback |
| **Smart Moms Canada**  [128] | SmartMoms Canada mobile app (Android and iOs) | Developed/adapted | FitBit Charge 2 Fitness Tracker + BodyTrace Scale | N/A | N/A | Educational content, self-monitoring of GWG, diet, PA, goal-setting, data driven feedback |
| **Smart Moms in WIC/ Healthy Beginnings**  [88,129] | Healthy Beginnings mobile app | Developed/adapted | Fitbit Alta + scale (Bodytrace) | N/A | tracked data automatically transmitted to clinician and participant dashboard | Self-monitoring of weight, PA, goals, automated prescriptive feedback from app, personalized feedback from mobile app, adaptive, prompts (motivation/success, alerts when outside recommended range) |
| **SpringMom**  [89,130] | Mobile/smartphone app (at least iOs 8.0 and Android 4.4) | Commercial | Body composition monitor and scale (Omron Healthcare Inc.) + Fitness tracker (Fitbit Inspire) | N/A | N/A | Educational content, personal advice based on data, feedback, self-monitoring of weight, PA, diet (meal photos), sleep |
| **StartSmart**  [131,142] | StartSmart mobile app | Developed | N/A | N/A | N/A | Screen for risk and protective factors, motivational interviewing in brief intervention, educational content |
| **STRIDE**  [90,139] | Hybrid – accessible via desktop or smartphone (iOs Android) | Developed/adapted | Physical activity tracker (wristband; Withings Activité Pop) + Scale (Withings Body Digital Scale) | N/A | Clinical portal | Real-time data from activity tracker and scale transmitted to mHealth site  goals, educational material |

Abbreviations: App, application; BCT, behaviour change technique; BP, blood pressure; GWG, gestational weight gain; HR, heart rate; iOs, internetwork operating system; N/A, not applicable; PA, physical activity; SMART, specific, measurable, achievable, relevant, time-bound; Wi-Fi, wireless fidelity
